# Psychosocial job exposure and risk of coronary artery calcification

**DOI:** 10.1371/journal.pone.0252192

**Published:** 2021-05-25

**Authors:** Helena Eriksson, Kjell Torén, Annika Rosengren, Eva Andersson, Mia Söderberg

**Affiliations:** 1 Department of Occupational and Environmental Medicine, Sahlgrenska University Hospital, Gothenburg, Sweden; 2 Occupational and Environmental Medicine, School of Public Health and Community Medicine, Institute of Medicine, Sahlgrenska Academy, University of Gothenburg, Gothenburg, Sweden; 3 Department of Molecular and Clinical Medicine, Institute of Medicine, Sahlgrenska Academy, University of Gothenburg, Gothenburg, Sweden; Nagoya University, JAPAN

## Abstract

**Purpose:**

The aim was to examine potential associations between psychosocial job exposures, evaluated with the Job Demand-Control-model, and presence of coronary artery calcium.

**Methods:**

We performed a cross-sectional study using the Swedish CArdioPulmonary bioImage Study,(SCAPIS)pilot study. Coronary artery calcium was assessed through computed tomography of the coronary arteries and with coronary artery scoring, CACS. Main outcome was CACS ≥100 compared to CACS 0. Job demand and control was analysed according to the standard categorization of the two variables into: high strain, active, passive and low strain (reference). Associations between these variables and CACS were calculated with prevalence ratios (PR) using Cox regression with robust variance, 95% confidence intervals (CI) and adjusted for age, smoking, education, socioeconomic area and metabolic syndrome.

**Results:**

In total 777 participants were used in our analyses, for which 20% of the men and 5% of the women had CACS ≥100, respectively. The PR of having CACS ≥100 was non-significantly elevated for men in high strain jobs 1.54 (95% CI 0.88–2.69) and in active jobs 1.67 (95% CI 0.92–3.06), adjusted for covariates. For women there was no association between exposure to high strain and having CACS ≥100 PR 1.02 (95% CI 0.24–4.31). Among women reporting passive job, the PR was non-significantly elevated, 2.40 (95% CI 0.83–6.92), adjusted for covariates.

**Conclusion:**

The statistical power of the study was limited, but our results suggests the possibility that exposure to a high strain or an active job situation may increase the risk of CACS in men, while in women, it may rather be exposure to passive job.

## Introduction

Widespread evidence relates adverse psychosocial work conditions to coronary heart disease (CHD) [1–4]. The most influential and evaluated psychosocial exposure model is the Job Demand-Control, JDC-model [5, 6], evaluating work volume and buffering effects from job control. High strain (high demand-low control) is the condition most associated with adverse health effects, while active work (high demand-high control) is considered as challenging, but stimulating and with the positive effects from high work control. Passive work (low demand-low control) is regarded as a low stress, but also as a work situation with low stimulation. Low strain, consisting of low demand and high control, is most strongly related to both physical and mental wellbeing and generally used as reference in psychosocial modelling. A large amount of literature links high strain, to CHD [2–4, 7]. For example, a recent meta-analysis of 13 studies concluded that there was an association between high strain and CHD, hazard ratio 1.23 (95% confidence interval, CI 1.10–1.37) [8]. As detrimental exposures in modern work life are increasingly dominated by psychosocial dimensions, health effects from these exposures are of increasing relevance to public health [9, 10]. Despite substantial evidence that relates job stressors to CHD, intermediary mechanisms are still unknown. Associations between high strain and hypertension or blood lipids have been investigated, but effects are mostly small and non-significant [4, 11–13].

Coronary artery calcification (CAC) is part of the atherosclerosis process. CAC develops through inflammatory mechanisms and general influence from mineral metabolism factors in the coronary arteries, in brief [14]. CAC increases with increasing age and generally develops at a later age among women compared to men [15]. The quantification/scoring of CAC can be made in different ways, Agatston score, Calcium volume score, Calcium mass score and Calcium density score [15].

CAC is an established predictor for CHD [16, 17]. A calcium score of zero is strongly associated with lack of coronary events over the subsequent 5 years in individuals without coronary symptoms [15]. Still, few studies have investigated the association between stressful work environments and CAC. In the longitudinal Coronary Artery Risk Development in Young Adults study, the analyses could not find any associations of CAC to job strain variables [18]. However, blue-collar workers in that study displayed a tendency of a higher prevalence of positive CAC compared to workers in other occupations [18]. Similarly, in a cross-sectional study of 1,849 subjects, no association between CAC and job strain was found [19].

The aim of this study was to examine potential associations between psychosocial job exposures, evaluated with the JDC-model and presence of coronary artery calcium, using data from the Swedish CArdioPulmonary bioImage Study (SCAPIS) pilot study.

## Methods

The SCAPIS study is a comprehensive research project which uses new imaging technologies and epidemiological analyses to extensively investigate CHD risk factors in women and men aged 50 to 64 years. The results will be used to improve risk prediction of cardiovascular diseases [20].

We used cross-sectional data from the SCAPIS pilot study for the present investigation. Participants, 2,243 men and women registered as residents in Gothenburg aged 50–64 years in 2012, were randomly selected, from either low or high socioeconomic geographical areas, and invited to participate in the study. A total of 1,111 accepted study participation (response rate = 49.5%) and were examined with blood samples, and computed tomography of the lungs and coronary arteries including coronary artery scoring and a questionnaire.

### Coronary artery calcification

CAC was estimated using a multi-slice computed tomography scanner (Siemens, Somatom Definition Flash, Siemens Medical Solution, Forchheim, Germany) [21]. Imaging and analyses were performed by using a calcium scoring protocol according to international standards [20–23]. A subset of the subjects (n = 84) were imaged using a 100 kV protocol, CAC from these subjects has been recalculated to the standard 120 kV [24]). The calcium content in each coronary artery was measured, summed and quantified using the Agatston score [23]. An Agatston score (CACS) ≥100 has been recognised to be significantly associated with an increased CHD risk [25]. CACS <100 has been reported as a low risk score [16]. In this study we compared CACS 0 with CACS ≥100 as our main outcome, as have been done previously [21, 25]. CAC was not measured in the case of presence of cardiac stent or previous by-pass surgery (n = 38).

### Psychosocial work variables

Job demand and control were estimated with the Swedish version of Karasek & Theorell’s Job Content Questionnaire, The Swedish Demand-Control-Support Questionnaire (DCSQ) [26]. The demand and control items were positively inverted so that high scores were equivalent to high demands or high control and then summed up separately. Since job demand and control were analysed using sum scores, subjects with <50% missing items received imputed values, mean scores of the remaining items in each variable were imputed on individual level. Subjects lacking ≥50% filled-in items per each variable were excluded (n = 289). Each variable was then dichotomized into high or low by the median values of the distributions. The dichotomized variables were combined into the following categories: ***high strain job (***high demand-low control), ***active job*** (high demand-high control), ***passive job*** (low demand-low control) and ***low strain job*** (low demand-high control) and the participants were allocated into these categories according to their job demand and control scores.

### Other variables

The questionnaire also recorded smoking habits, occupation, weight, height and marital status. The participants were classified as having metabolic syndrome or not, using the criteria for clinical diagnosis of the metabolic syndrome according to a statement from the American Heart Association (AHA) and the National Heart, Lung, and Blood Institute (NHLBI) [27]. Presence of any three of the following five parameters were regarded as constitution of the metabolic syndrome: elevated waist circumference, ≥88 cm in women and ≥102 cm in men; elevated triglycerides, ≥1,7 mmol/l or drug treatment for elevated triglycerides; reduced HDL cholesterol, <1,3 mmol/l in women and <1,03 mmol/l in men or treatment with statins; elevated blood pressure, systolic blood pressure ≥130 or diastolic blood pressure ≥85 or hypertensive drug treatment; elevated fasting glucose, ≥5,5 mmol/l or treatment with antidiabetic drugs or insulin. Subjects with missing data for these parameters were excluded (n = 7), leaving 777 subjects for the study.

### Statistical analyses

The participants were divided in three groups according to their CACS; CACS 0, CACS 1–99 and CACS ≥100, Table 1. Descriptive statistics are presented as percentages within each group or mean with standard deviation, Table 1. For covariates, except for age, significant difference (p<0.05) was tested between the reference group (CACS = 0) and the other groups, using Pearson’s chi-squared test and Fisher´s exact test for the women due to few cases. Associations between psychosocial work variables and CACS were calculated with prevalence ratios (PR), using Cox regression with constant time at risk and robust variance and 95% confidence intervals (CI) [28]. We used this method as cases constituted more than 10% of the participants. The groups CACS 1–99 and CACS ≥100, respectively, were compared to CACS 0. The following covariates were used: Age was entered as a continuous variable in years. Smoking status was divided into two categories; ever smoker versus never smoker (reference). No university education was compared to completed university education and living in a low socioeconomic area was compared to living in a high socioeconomic area. Metabolic syndrome was divided into present or not. Correlations between covariates, the four categories of job demand-control and CACS groups were checked with correlation coefficients, which were all <0.3 except gender and CACS (r = -0.33) and we show stratified analyses for gender.

**Table 1:** Characteristics of subjects by gender and coronary calcium score (CACS).

	Men N = 384			Women N = 393		
	CACS 0	CACS 1–99	CACS ≥100	CACS 0	CACS 1–99	CACS ≥100
**N (%/gender)**	170 (44%)	137 (36%)	77 (20%)	293 (74%)	82 (21%)	18 (5%)
**Mean age (SD)**	56.2 (4.4)	57.4 (3.9)	59.3 (4.0)	56.6 (4.1)	58.8 (3.9)	61.5 (2.9)
**Covariates**						
**Low strain job N (col %)**	31 (18%)	19 (14%)	9 (12%)	74 (25%)	22 (27%)	4 (22%)
**High strain job N (col %)**	43 (25%)	39 (28%)	29 (38%)*	61 (21%)	15 (18%)	3 (17%)
**Active job N (col %)**	21 (12%)	21 (15%)	16 (21%)	55 (19%)	16 (20%)	2 (11%)
**Passive job N (col %)**	75 (44%)	58 (42%)	23 (30%)*	103 (35%)	29 (35%)	9 (50%)
**No university N (col %)**	103 (61%)	91 (66%)	48 (62%)	140 (48%)	46 (56%)	15 (83%)*
**Ever smoker N (col %)**	70 (41%)	78 (57%)*	54 (70%)*	152 (52%)	49 (60%)	12 (67%)
**Socioeconomic area N (col %)**	63 (37%)	56 (41%)	37 (48%)	105 (36%)	31 (38%)	13 (72%)*
**Metabolic syndrome** **N (col %)**	36 (21%)	53 (39%)*	40 (52%)*	68 (23%)	30 (37%)*	7 (39%)

col %—% in each column that is in every group of CACS stratified for gender.

* represents that the frequency of the covariate differed significantly in these CACS-groups compared to CACS = 0.

Two models were calculated; one model with adjustments for age and a second model with adjustment for age, smoking, education, socioeconomic area and metabolic syndrome (all p<0.05 when adding one by one to the first model for CACS ≥100 and all but socioeconomic area p<0.05 for CACS 1–99). If the model was applied on all participants, adjustment for gender was done. Marital status was also tested but when adding that to the first model; p>0.25 for CACS ≥100 so marital status was omitted from further analyses. The statistical analyses were performed with the statistical software package SAS Enterprise guide version 7.1 and SAS 9.4 (SAS Institute, Cary, NC, USA).

### Ethics approval and consent to participate

Informed written consent was obtained from all the individuals who participated in the study. All procedures involving human participants were in accordance with the ethical standards of the institutional and/or national research committee and conformed to the 1964 Helsinki declaration and its later amendments or comparable ethical standards. Ethical approval for the study was granted by the Ethical Review Board of Gothenburg and the Ethical Review Board of Umeå, Sweden, permit no. 2010-228-31M.

## Results

In total, 777 participants had complete data on job demand-control and chosen covariates. Among participants 20% of the men and 5% of the women had CACS ≥100, respectively, Table 1. Men with CACS ≥100 were more frequently ever smokers, 70% (p = <0.0001), had a metabolic syndrome, 52% (p = <0.0001) and more often a high strain job 38%, (p = <0.048), Table 1. While women with CACS≥100 more frequently reported no university education or living in a low status socioeconomic area 83% (p = 0.003) and 72%, (p = 0.004), respectively, Table 1. Women with CACS 1–99 more often had a metabolic syndrome, 37% (p = 0.022). Table 1. None of the adjustment factors were significantly related to high strain jobs, among neither men nor women.

For women there was no association between exposure to high strain and having CACS ≥100 PR 1.02 (95% CI 0.24–4.31), adjusted for age, education, smoking, socioeconomic area and metabolic syndrome, Table 2. For women reporting passive job, the risk of having CACS ≥100 was PR 2.40 (95% CI 0.83–6.92). Male participants reporting high strain job had a risk PR 1.54 (95% CI 0.88–2.69) of having CACS ≥100 and those reporting active job; PR 1.67 (95% CI 0.92–3.06), respectively, Table 2.

**Table 2:** Prevalence Ratios (PR) with 95% confidence intervals (CI) between exposure to job demand-control and CACS.

	CACS = 1–99 compared to CACS = 0		CACS≥100 compared to CACS = 0	
	PR (95% CI) age-adjusted*	PR (95% CI) adjusted**	PR (95% CI) age-adjusted*	PR (95% CI) adjusted**
**All subjects, N**	219–463	219–463	95–463	95–463
**High strain job**	1.00 (0.72–1.39)	1.01 (0.72–1.41)	1.35 (0.79–2.31)	1.47 (0.86–2.51)
**Active job**	1.06 (0.74–1.51)	1.02 (0.72–1.44)	1.21 (0.66–2.20)	1.32 (0.73–2.39)
**Passive job**	1.01 (0.75–1.37)	1.06 (0.78–1.45)	1.18 (0.68–2.05)	1.49 (0.84–2.63)
**Women, N**	82–293	82–293	18–293	18–293
**High strain job**	0.79 (0.45–1.40)	0.83 (0.46–1.50)	0.75 (0.17–3.21)	1.02 (0.24–4.31)
**Active job**	0.92 (0.53–1.59)	0.93 (0.54–1.57)	0.59 (0.12–2.80)	0.70 (0.16–3.02)
**Passive job**	0.93 (0.58–1.50)	1.06 (0.64–1.73)	1.44 (0.49–4.20)	2.40 (0.83–6.92)
**Men, N**	137–170	137–170	77–170	77–170
**High strain job**	1.18 (0.78–1.80)	1.21 (0.79–1.84)	1.56 (0.86–2.83)	1.54 (0.88–2.69)
**Active job**	1.23 (0.77–1.97)	1.18 (0.75–1.86)	1.51 (0.78–2.95)	1.67 (0.92–3.06)
**Passive job**	1.12 (0.75–1.67)	1.16 (0.78–1.74)	1.09 (0.58–2.06)	1.27 (0.68–2.37)

*all subjects adjusted for age and gender.

**adjusted for age, education, smoking, socioeconomic area and metabolic syndrome, all subjects also adjusted for gender.

Groups of coronary calcium score (CACS) analysed in relation to exposure for job demand-control. High strain job, active job and passive job are compared with low strain job with prevalence ratios (PR) and 95% confidence intervals (CI).

When stratifying men into those with or without metabolic syndrome the risk of CACS≥100 among men with high strain job but no metabolic syndrome was PR 1.42 (95% CI 0.58–3.44) and among men with high strain job and presence of metabolic syndrome PR 1.62 (95% CI 0.80–3.30) (both adjusted for the significant covariates age and ever-smoking).

## Discussion

The present cross-sectional study suggests the possibility that established adverse psychosocial job exposure, high strain job, but also active job might potentially increase the risk of coronary artery calcium in men, however, none of the prevalence ratios were significant. Among women, high strain did not increase the risk of CAC. Although non-significant, the results suggested increased risks of CAC in passive work environments for women.

Previous studies have not detected any associations between JDC-model variables and CAC, although these are not wholly comparable to our study since they were conducted in young [18] or high risk subjects [19]. For example, in the longitudinal Coronary Artery Risk Development in Young Adults study, aged 18–30 at baseline, CAC was measured in 3,695 participants at 15 and 20 years of follow-up [18]. Neither the single variables low control or high psychological demands, or combined into high strain (high demands-low control) were associated with CAC. Since CAC was measured when the subjects were 38–50 years old, lack of associations could be due to the relatively young age as CAC relates to an older age [16].

We calculated prevalence ratios between psychosocial work variables and CACS by using COX regression with robust variance. We did not calculate odds ratios since they can overestimate risks when risks are above 10–15%. We adjusted for the metabolic syndrome, it has been related to an increased frequency of CAC [29], which we also found especially among men. Even though the metabolic syndrome could be interpreted as a mediator, since job strain has been related to an increased risk of metabolic syndrome [30], temporal assumptions could not be met for a mediator analysis in this study, due to the cross-sectional design. However, when stratifying for the metabolic syndrome, the risk was not different for developing CAC among men without the metabolic syndrome, but this could be due to the lack of power.

CAC is also more frequent among men and older age, as reported in a previous prospective cohort study where they investigated the amount of CAC in a cohort of 6,814 participants 45 to 84 years of age without clinical cardiovascular disease [31].

Even though none of the results were significant, it seems that for women, it was rather passive job exposure that increased the risk of developing CAC, while for men it was high strain and an active job that increased the risk. The results did not show that high strain increased the risk for CAC among women, but this could be due to the age of the participants in the study and low power. The subjects in this study were aged 50–65 years of age. Women generally have a later development of coronary heart disease and CAC compared to men [32, 33].

There are advantages with this study. The subjects are derived from the general population and from different socioeconomic areas. The exposure, job strain was estimated through a validated questionnaire and the outcome, CAC, was measured with a computed tomography investigation, which increases the validity of the results. We also had information on covariates such as smoking, gender and metabolic syndrome.

However, there are also limitations, the study included relatively few participants. We consequently also had a lack of statistical power. The low power was relevant in the gender stratified analyses, as only 3 women reported high strain and displayed high CAC-scores. The study was also limited to certain ages, which is a limitation especially for women since they usually develop CAC at a later age. We lacked information on physical activity and could not adjust for this covariate.

## Conclusions

Our results suggests the possibility that exposure for high strain job or active job may increase the risk of CAC in men, but in women, it could rather be exposure for passive job that increases the risk. However, there was a lack of power in the study.
