# Supplementary material for: Psychosocial job exposure and risk of coronary artery calcification
Source: PLoS One. 2021 May 25;16(5):e0252192. doi: 10.1371/journal.pone.0252192 (PMC8148350; doi:10.1371/journal.pone.0252192)
Supplement: Attachment — Submitted filename: Response to reviewers_coronarycalciumdec20_helenaeriksson.docx [file pone.0252192.s001.docx]

Response to reviewers

*1. Please ensure that your manuscript meets PLOS ONE's style requirements, including those for file naming. The PLOS ONE style templates can be found at*

[*https://journals.plos.org/plosone/s/file?id=wjVg/PLOSOne_formatting_sample_main_body.pdf*](https://journals.plos.org/plosone/s/file?id=wjVg/PLOSOne_formatting_sample_main_body.pdf) *and*

[*https://journals.plos.org/plosone/s/file?id=ba62/PLOSOne_formatting_sample_title_authors_affiliations.pdf*](https://journals.plos.org/plosone/s/file?id=ba62/PLOSOne_formatting_sample_title_authors_affiliations.pdf)

Thank you for your reminder. The table titles and some of the text in the tables has been changed to bold letters. The affiliations have been adjusted.

*2. Please address the following:
- Please ensure you have thoroughly discussed all potential limitations of this study within the Discussion section, including the potential impact of confounding factors.*

Thank you for your comment. We have now further developed the limitation section. Regarding the influence of confounding factors, we had information on smoking, gender, blood lipids and blood pressure which we adjust for and present in the manuscript, before and after adjustment. We lacked information on physical activity which could be considered a confounder, we have added this to the limitation section.

*Please include additional information regarding the survey or questionnaire used in the study and ensure that you have provided sufficient details that others could replicate the analyses. For instance, if you developed a questionnaire as part of this study and it is not under a copyright more restrictive than CC-BY, please include a copy, in both the original language and English, as Supporting Information.*

The participants in SCAPIS filled-in a questionnaire covering many different domains, for example smoking and job strain. The questions regarding job strain constitutes an established instrument that has been previously validated in a study by Sanne et al 2003. We will upload the reference with the validation study and where you also can see the questions.

*We note that you have indicated that data from this study are available upon request. PLOS only allows data to be available upon request if there are legal or ethical restrictions on sharing data publicly. For information on unacceptable data access restrictions, please see* [*http://journals.plos.org/plosone/s/data-availability#loc-unacceptable-data-access-restrictions*](http://journals.plos.org/plosone/s/data-availability#loc-unacceptable-data-access-restrictions).

*In your revised cover letter, please address the following prompts:*

*a) If there are ethical or legal restrictions on sharing a de-identified data set, please explain them in detail (e.g., data contain potentially identifying or sensitive patient information) and who has imposed them (e.g., an ethics committee). Please also provide contact information for a data access committee, ethics committee, or other institutional body to which data requests may be sent.*

*b) If there are no restrictions, please upload the minimal anonymized data set necessary to replicate your study findings as either Supporting Information files or to a stable, public repository and provide us with the relevant URLs, DOIs, or accession numbers. Please see* [*http://www.bmj.com/content/340/bmj.c181.long*](http://www.bmj.com/content/340/bmj.c181.long) *for guidelines on how to de-identify and prepare clinical data for publication. For a list of acceptable repositories, please see* [*http://journals.plos.org/plosone/s/data-availability#loc-recommended-repositories*](http://journals.plos.org/plosone/s/data-availability#loc-recommended-repositories)*.*

*We will update your Data Availability statement on your behalf to reflect the information you provide.*

Data cannot be shared publicly because information about health and symptoms are regarded as sensitive information, and when sharing such data there has to be an approval from a Swedish Ethical committee (according to Swedish law). However, anonymised data is available with an approval from an ethical review board. For data requests, contact: [scapis@scapis.org](mailto:scapis@scapis.org). The name of the data set is SCAPIS-pilot. We will also add this information to the cover letter.

*4. We noted in your submission details that a portion of your manuscript may have been presented or published elsewhere.*

*[An early draft of the manuscript has been published in paper, but not digitally, in a thesis, the thesis of the corresponding author. It is not possible to upload the whole thesis. I can send you one by mail if you like? ]*

*Please clarify whether this [conference proceeding or publication] was peer-reviewed and formally published. If this work was previously peer-reviewed and published, in the cover letter please provide the reason that this work does not constitute dual publication and should be included in the current manuscript.*

We apologies for not being clear on this point. The thesis is the first author’s PhD-thesis/doctoral dissertation and was only presented orally during the viva, i.e. the "dissertation defence” but it has not been peer-reviewed nor formally published, only printed in paper in a limited amount as part of the PhD-thesis.

*Reviewer #1: This ms reports results of analyses of the associations of established measures of stress at work (high job strain, active job and passive job) with coronary artery calcium score (CACS, an established measure of coronary atherosclerosis) in a sample of healthy Swedes on whom they also have measures of the metabolic syndrome (waist circumference, triglycerides, HDL cholesterol, blood pressure and fasting glucose). They report in men non-significant associations of high job strain and active job with higher prevalence ratios (PR) of CACS >100 and in women non-significant association of passive job with higher PR of CACS >100. There are several concerns regarding the statistical analyses the authors used that need to be addressed before a final decision can be reached regarding this ms.*

Thank you for the summary. Please let us know if the concerns regarding the statistical analyses are about the analyses done or those not done.

*Rather than only doing Cox regression analyses evaluating associations between the job stress measures with PR of CACS 1-99 or CACS > 100 with only age-adjustment or age-adjustment and adjustment for education, smoking, SES area and metabolic syndrome, they need to do more comprehensive analyses that take into account associations of the job stress and CACS measures with the variables they adjust for. In Table 1, for example, men with CACS >100 appear to have higher level of metabolic syndrome (52%) than men with CACs=0 (21%), they also have higher smoking rate (70%) than men with CACS=0(41%). There is no mention in the Results of whether these associations of CACS>100 with increased metabolic syndrome and smoking are statistically significant. It could also be informative if they carried out analyses of the associations between CACs and the components of the metabolic syndrome. That is, are some of the components of the metabolic syndrome – e.g. glucose, triglycerides and BP – accounting for the association of the metabolic syndrome with CACS>100.*
Thank you for your comments. Yes, we always want to know more, however the aim of the study was to analyze if exposure to job strain was associated with development of coronary artery calcium, CAC, not what factors that causes CAC. That is why we do not present these associations in the results, although in Methods we have presented whether the associations are significant: ”a second model with adjustment for age, smoking, education, socioeconomic area and metabolic syndrome (all p<0.05 when adding one by one to the first model for CACS ≥100 and all but socioeconomic area p<0.05 for CACS 1-99).” The study is also too small to go into details of the components of the metabolic syndrome. We hope to do that in the larger study.

*They also need to evaluate associations between the job stress measures and all the variables they adjust for in the regression analyses. If, for example, higher job strain is associated with the same factors—i.e., smoking and metabolic syndrome – that appear to be associated with CACS>100, it could be the case that there is a statistically significant indirect pathway from high job strain à metabolic syndrome (or some of its components) and smoking àincreased CACS. They do cite a paper (30) in the Discussion that found job strain associated with increased risk of metabolic syndrome. They also cite a paper in the Introduction (11, Nyborg et al.) that found increased job strain associated with increased diabetes, smoking, physical inactivity and obesity. A paper by Jensen et al. (Circ Cardiovasc Imaging Epub, July 14, 2020) found obesity associated with increased CAC and increased CHD and all cause mortality over a follow-up period.

They could test this pathway using structural equation modeling. If this indirect pathway from job stress indices to increased CACS is found significant, this paper would be making a far greater contribution to our understanding of the impact of increased stress at work on mechanisms involved in the development of coronary artery disease.*

Thank you for your comments. The aim of the study was to analyze if exposure to job demand-control was associated to development of coronary artery calcium. The standard procedure in this area of research is to establish association between work exposure and an outcome, and if that association remains when entering known covariates. Although we would think it would produce interesting results, illustrating possible pathways between stressful job exposure and health, in this cross-sectional data it is not possible since mediator analyses require the temporal aspect of x🡪M 🡪 y. The main SCAPIS cohort, from which the data used in this paper is the pilot data, will be a longitudinal study, where we hope to perform the suggested analyses in the future. We are currently in the process of initiating another study on the core cohort material where we aim to establish interactive effects from job exposure are markers for socioeconomic status, since it’s a much larger material and will take these insightful comments into consideration.

We have not presented data about correlations between the used covariates, we will add that. Gender was the one with highest correlation, r=-0.33 (both with Pearson and Spearman), and we present the results for both genders separately. All other covariates, outcome and four kinds of job stress was less correlated than r=0.3 to each other. We have made stratified analyses for metabolic syndrome, ever-smoking and socioeconomic area, respectively (not shown), among men and CACS>100 without finding any major changes between strata or in results. For women, there are too few cases.

*There is another measure of stress at work, effort/reward imbalance (ERI), that might be worth evaluating using the same approach (especially as expanded in line with concerns above).*

Thank you for your suggestion, unfortunately we do not have data on exposure to effort reward imbalance, the participants have not answered questions concerning effort/reward*.*

*Reviewer #2: The authors provide an interesting and potential important manuscript describing "psychosocial job exposure and risk of coronary artery calcification", The main issues concerning this paper are those concerning the potential associations between psychosocial exposures and coronary calcium.*

*There are some weak points that need to be addressed by the authors.

Major

1. The author should supplement CACS, the Swedish Demand-Control-Support Questionnaire, the specific evaluation criteria for calcification and working stress.*

The amount of coronary artery calcium is frequently quantified through the Agatston scoring method where calcium deposits areas are multiplied by a density factor estimated through a computed tomography investigation. The Agatston scoring method is described in reference 23, which we will supplement (Agatston AS, Janowitz WR, Hildner FJ, Zusmer NR, Viamonte M, Jr., Detrano R. Quantification of coronary artery calcium using ultrafast computed tomography. J Am Coll Cardiol. 1990;15:827-32). We will also supplement reference 26 where one can see the questions that we have used (Sanne B, Torp S, Mykletun A, Dahl AA. The Swedish Demand-Control-Support Questionnaire (DCSQ): factor structure, item analyses, and internal consistency in a large population. Scand J Public Health. 2005;33:166-74).

*2. I think the direction chosen in this paper is very good. But the conclusion is bad supported by the data in this paper. First the conclusion say “exposure to a high strain or active job situation could potentially increase the risk of CACS in men”, We don't know if these people are representative of people in social groups who work under different pressures. Second in 384 men, there are 59 people in low strain job, 58 people in active job,111 people in high strain job,156 people in passive job, We can see that most people live in stressful jobs, so it is unscientific to conclude that stressed people have more calcification.*

# Thank you for your comment. According to the model theory, developed by Karasek and Theorell adverse health effects from work exposure is determined by the volume of work tasks (demand) and positive buffering effects from work control (Karasek R, Theorell T. Healthy work; stress, productivity and the reconstruction of working life. New York, N.Y: Basic Books; 1990). Thus the categorization of participants into: 1. Low strain job =low demands/high control 2. Active job=high demands/high control 3. Passive job=low demands/low control 4. High strain job=high demands/low control, creates a categorization of 4 different work situations. High demand combined with low control is regarded as the most detrimental work type, which has been established in a wide range of studies. Traditionally “active” work is conceived as intense but also stimulating and not hazardous as the workers influence over the work situation may lower adverse effects from high work volumes. Passive job, low demands-low control, has been conceived as either positive or negative in the first decades of research with this model, but in recent years there has been evidence that this more monotonous work situation may for example spill over to the leisure time, as workers with passive jobs tend to have less physical activity in their leisure time. Low strain (low demand-high control) is generally assigned as a reference and a preferred work situation as it entails low stress from high amounts of work tasks, but also high freedom within one’s job.
