# Supplementary material for: Psychosocial job exposure and risk of coronary artery calcification
Source: PLoS One. 2021 May 25;16(5):e0252192. doi: 10.1371/journal.pone.0252192 (PMC8148350; doi:10.1371/journal.pone.0252192)
Supplement: Attachment — Submitted filename: Response-PONE-D-20-20980_R1.doc [file pone.0252192.s002.doc]

Dear author

Thank you very much for providing the supplement CACS and explain the categorization of participants. This paper may highlight the psychological factor on vascular calcification, which may notice people care for the work stress.
