# Supplementary material for: Psychosocial job exposure and risk of coronary artery calcification
Source: PLoS One. 2021 May 25;16(5):e0252192. doi: 10.1371/journal.pone.0252192 (PMC8148350; doi:10.1371/journal.pone.0252192)
Supplement: Attachment — Submitted filename: resprevjan19herikssoncacsubm.docx [file pone.0252192.s003.docx]

18^th^ of January 2021

Gothenburg

Response to reviewers

*We have marked all of our changes in yellow in the manuscript.*

*In the Abstract we have made some changes to make it clearer. Also, in Introduction first paragraph, we have added some text regarding the job strain model to try to make it more clear for the reader.*

Reviewer #1: The authors have not responded adequately to my concerns regarding the original version of this ms. With a sample of 384 men and 393 women, there is ample power for them to conduct and report results of exploratory tests of associations between the variables adjusted for in the analyses of associations between job stress measures and CACS and the job stress measures and CACS levels themselves.

*Thank you for your recommendations on further analyses. We regret if our methodology has not been clear enough to illustrate that in this study the participants with established CAC are divided into four exposure groups, depending on their reporting of high or low job demand or job control, hence, there are sometimes few cases in each exposure group. If one further divides the participants according to for example presence of the metabolic syndrome there will be even less cases in each exposure group. Please see the table below where we have tried to clarify this. As the amount of cases are few, the statistical power would be too low for further analysis of potential associations.*

*Cases with CACS≥100 among men by job type and metabolic syndrome. Each of the other job types are compared with low strain job in the analyses.*

|  | *Low strain job* | *High strain job* | *Active job* | *Passive job* | *Total* |
| --- | --- | --- | --- | --- | --- |
| *No metabolic syndrome* | *5* | *11* | *9* | *12* | *37* |
| *Metabolic syndrome* | *4* | *18* | *7* | *11* | *40* |
| *Total* | *9* | *29* | *16* | *23* | *77* |

They do note in Methods (lines 153-156) that the covariates (age, smoking, education, socioeconomic area and metabolic syndrome) were “all P< 0.05 when adding one by one to the first model for CACS > 100..” If I interpret this correctly, this means that all these covariates were significantly associated significantly with severe CACS > 100. There is no reason they should not report these associations in a table – or indicate in Table 1 that the 52% of men with CACS >100 is significantly different from the 21% of men with metabolic syndrome in those with CACS of 0, with similar indicates for the other covariates.

*Thank you, we have now marked these significant differences in table 1, we have also added explanatory text to table 1 and text in Methods/Statistical analyses, third sentence.*

In addition to these presumed associations of the covaried risk factors with CACS, attention also needs to be given to their associations with the job stress measures. If, as I noted in my review of the original ms, there is a significant association of high job strain with high levels of metabolic syndrome, it would suggest the possibility, even if they do not provide a direct test of metabolic syndrome as a mediator, that the increased CACS in men with high job strain may be the result of their higher level of metabolic syndrome.

*For several reasons, please also see our first answer in this letter and further down, we cannot do the suggested analyses, but we can present a stratified analysis regarding metabolic syndrome which hopefully provides the reviewer with some answers. In Results, last section, page 10 we have now presented the results for stratification of men in to those with the metabolic syndrome and those without it, the risk of CACS≥100 among men with high strain job but no metabolic syndrome was PR 1.42 (95% CI 0.58-3.44) and among men with high strain job and metabolic syndrome was PR 1.62 (95% CI 0.80-3.30) (both adjusted for the significant covariates age and ever-smoking).*

Given that prior research (ref 30) has found an association between high job strain and metabolic syndrome, there is every reason to expect they will be able to replicate that finding in the current study. I don’t disagree with their point that using SEM to assess possible pathways is now considered to require the temporal aspect to be conclusive with respect to causation. It might still be informative, however, even with the current cross-sectional data, to perform SEM pathway analysis, as an exploratory way of seeing if there might be a pathway from job strain to higher CACS via metabolic syndrome. Even though it could not be considered definitive (which the authors would note in a revised ms), if such an SEM path analysis did identify a statistically significant indirect pathway from high job strain to high metabolic syndrome (or other covariates or metabolic syndrome components per below) and from there to high CACS, it would be encouraging that, despite their failure to find a significant association between job strain and CACS, there is reason to continue to pursue ongoing research (including longitudinal) to evaluate job stress measures as risk factors for CACS (and CVD events). Moreover, finding such a significant indirect pathway could also help identify intervention targets in men in high strain jobs – e.g., if there’s a significant indirect pathway from job strain via smoking to CACS, that would suggest stopping smoking as an important preventive approach in men in high strain jobs.

Speaking of exploratory analyses, there is no reason, given a total sample of 777 men and women, for them to assert that “the study is too small to go into details of the components of the metabolic syndrome.” If the metabolic syndrome itself (and other covariates) are significantly associated with CACS (and job stress measures?), it should be possible to determine whether the association of metabolic syndrome itself with CADS is due to one or more specific components of the metabolic syndrome.

*Thank you for your comments and your interest in the complex possible pathways between psychosocial stressors and cardiovascular outcomes. We provided our limitations in terms of lacking statistical power under the first paragraph of your revision requests.*

*Regarding adding analyses of metabolic syndrome: There is throughout the literature established relationships between adverse psychosocial job exposure and coronary heart disease, such as myocardial infarction. However, the intermediary pathways are not known. The aim of this study was to study associations between exposure to work related stress and CAC, which is a plausible pathway in the disease chain between job stressors and CHD. We feel that since the focused outcome is already an intermediary variable, examining yet another intermediary variable and its relationship to CAC, would make the study less focused, especially since this is a cross-sectional study and causality cannot be established between CAC and metabolic syndrome. Furthermore, in occupational medicine it is common to analyse the job exposure, and the strength of that association when adjusting for several covariates, without reporting the associations of the covariates, as they are not the focus or the variables of interest.*

They conclude that “Our results indicate that exposure for high strain job or active job could increase the risk of CAC in men, but in women, it could rather be exposure for passive job that increases the risk. However, there was a lack of power in the study.” Given that the associations of job stress measures with CACS in men and women were not statistically significant, I believe it would behoove the authors to perform exploratory analyses as outlined in my comments above to take maximum advantage of the extensive data they have on this sample of 384 men and 393 women.

*Please see our previous answers above regarding power. The power is based on the amount of cases, who when divided into the four different psychosocial job categories, are few in several cells.*

*The SCAPIS-pilot was performed to check all details in the large SCAPIS protocol but also to have data to predict selection bias from participation rates in different socioeconomic geographical areas (Björk et al. Scand J Public Health 2017;45(Suppl 17):45-49). In order to do that low and high socioeconomic areas were studied. We believed it was interesting to study job strain within a span of different job experiences as they came from both low and high socioeconomic areas, even with a limited population.*

Reviewer #2: Dear author

Thank you very much for providing the supplement CACS and explain the categorization of participants. This paper may highlight the psychological factor on vascular calcification, which may notice people care for the work stress.

*Thank you for feedback!*

7. PLOS authors have the option to publish the peer review history of their article ([what does this mean?](https://journals.plos.org/plosone/s/editorial-and-peer-review-process#loc-peer-review-history)). If published, this will include your full peer review and any attached files.


If you choose “no”, your identity will remain anonymous but your review may still be made public.


**Do you want your identity to be public for this peer review?** For information about this choice, including consent withdrawal, please see our [Privacy Policy](https://www.plos.org/privacy-policy).

Reviewer #1: No

Reviewer #2: No
