# Supplementary material for: Psychosocial job exposure and risk of coronary artery calcification
Source: PLoS One. 2021 May 25;16(5):e0252192. doi: 10.1371/journal.pone.0252192 (PMC8148350; doi:10.1371/journal.pone.0252192)
Supplement: Attachment — Submitted filename: resprev3mars21cacherikssonsubmit.docx [file pone.0252192.s004.docx]

Response to reviewers

3^rd^ of March 2021

*We have marked all of our changes in yellow in the manuscript.*

Reviewer #1: The authors have responded to my request to include indication of significant differences comparing CACS 1-99 and >100 groups with the CACs 0 group with respect to job stress measures, ever smoker, SES area, and Metabolic Syndrome measures in Table 1. And it is interesting to observe that in men only ever smoker and MetSyn are higher in CACS 1-99 and CACS >100 than CACS 0, and in women only No University and SES area are higher in CACS >100 and MetSyn is higher in CACS 1-99, but not CACS >100 – likely due to small N (7) in that group. They need to include these statistically significant differences in the Results section.

*We have now included this in the results section, first paragraph. We have, however, refrained to add further suggested analyses as this is not a part of our study aim, which focus on work exposure and health.*

Despite the fact that none of the tests of associations between job stress and other measures with CACS were significant in either men or women, they still conclude that, despite “a lack of power in the study,” “Our results indicate that exposure for high strain job or active job could increase the risk of CAC in men, but in women, it could rather be exposure for passive job that increases the risk.”

*Thank you for your comment, we have moderated the conclusion*

Given this lack of significant effects for the job stress measures, a strong case can be made that they should undertake exploratory analyses in this sample of 777 healthy middle-aged men and women to evaluate associations between job stress measures and other factors they find associated with increased CACS – i.e., ever smoker and MetSyn in men and No University, SES area and MetSyn in women – to determine whether these other factors that are associated with CACS and, therefore, at least potential mediators of effects of job stress on CACS, are increased in men and women with high job stress. If these potential mediators are significantly increased in men and women with high levels of job stress measures, it would at least enable the authors to suggest – even if they do not perform SEM path analyses, that these factors (especially ever smoked and MetSyn) could be mediators of associations between job stress and CACS.

*We have now checked this and none of the adjustment factors was significantly related to high strain jobs, among neither men nor women. However, we will not include this in the results section, since this is not our research question.*

A similar argument can be made for them to perform exploratory analyses to determine whether it is possible to identify specific components of the MetSyn that are accounting for the association between MetSyn and increased CACS in both men and women, and, if so, whether these MetSyn components are also elevated in men and/or women with high levels of job stress.

*This study was not an exploratory study and our research question was to to examine associations between psychosocial job exposures and presence of coronary artery calcium.*

*We do not believe that it is correct to set up a new research question within the same study when finding out that we have insignificant results. Also, as previously said, we do not have enough statistical power to do the suggested analyses.*

It is not clear how doing stratified analyses of job stress associations with CACS in men with MetSyn vs men with no MetSyn – all nonsignificant – addresses the question of mediation of job stress impact on CACS.

*Thank you for your comment. We agree that the metabolic syndrome can be a factor that partly explains the development of CAC, that is why we also have stratified for the metabolic syndrome not only adjusted for it. The stratified analyses may show that it is not only subjects with the metabolic syndrome that seems to be at higher risk of CAC in high strain jobs.*

Reviewer #2: Dear author

The paper entitled with Psychosocial job exposure and risk of coronary artery calcification show an interesting study to identify psychological stress on CVD disease, the detail mechanism for calcification need to further explore, but this phenomenon need our of us to notice.

Thank you very much to made a meaningful research.

*We are very thankful for your feedback*
