# Supplementary material for: Psychosocial job exposure and risk of coronary artery calcification
Source: PLoS One. 2021 May 25;16(5):e0252192. doi: 10.1371/journal.pone.0252192 (PMC8148350; doi:10.1371/journal.pone.0252192)
Supplement: Attachment — Submitted filename: resprevapr21cacserikssonsubm.docx [file pone.0252192.s005.docx]

*29^th^ of April 2021, Gothenburg*

*Response to reviewers.*

*We have marked all of our changes in yellow in the manuscript.*

PLOS ONE

Journal Requirements:

Please review your reference list to ensure that it is complete and correct. If you have cited papers that have been retracted, please include the rationale for doing so in the manuscript text, or remove these references and replace them with relevant current references. Any changes to the reference list should be mentioned in the rebuttal letter that accompanies your revised manuscript. If you need to cite a retracted article, indicate the article’s retracted status in the References list and also include a citation and full reference for the retraction notice.

*Thank you for your comment, regarding reference 27, we have now added “Corrected version” after the reference. We have also translated reference 4, 9 and 10 to English.*


[Note: HTML markup is below. Please do not edit.]

Reviewers' comments:

Reviewer's Responses to Questions

**Comments to the Author**

Reviewer #1: In this R3 draft the authors have made some constructive changes in response to my earlier reviews, but there are still some areas where improvement would be good.
They have included some reference to the univariate Fischer’s exact tests of differences in Table 1’s work variables and their chosen covariates in the first paragraph of the Results section, but I believe they need to include more details – i.e., in men with CACS >100 the 38% percent with high strain job was NOT significantly different from the 25% in men with CACS of 0. In contrast, Table 1 shows that in men with CASC > 100 or 1-99 both % Ever smoker and Metabolic syndrome were significantly elevated compared to men with CACS of 0; and in women those with CACS >100 had significantly higher % with No University and living in Low SES area than those with CADS 0, and those with CACS 1-99 had significantly higher % with metabolic syndrome. The higher % in passive work conditions in those women with CACS > 100 is NOT significant. I believe that the significant associations between CACS levels and % with Metabolic syndrome and Ever smoker in men and between CACS levels and No university and low status SES area and Metabolic syndrome in women should be reported in more detail – e.g., 83% of women with CACS >100 had No university vs only 48% of women with CACS of 0, and give the P-values (e.g., P<0.05) for each significant one.

*We apologize for causing a misunderstanding. It is only the covariates metabolic syndrome, no university education, ever smoker and socioeconomic area that we have previously marked with an asterisk in table 1, if it differed significantly, we have not marked high strain job, passive job, active job or low strain job. The reason is that these are unadjusted results and can be misleading as we later in the manuscript present the prevalence ratios between CACS and passive/low/high strain and active job, which are adjusted and therefor more robust results. But we have now also presented in table 1 which of the job types that differed significantly. We have also adjusted the text about table 1 in the results section, first paragraph and added only significant results from table 1 in the text. But please note that the results in table 1 are descriptive and not adjusted.*

They say they have moderated the conclusion to take into account that none of the tests of associations between job stress and other measures with CACS were significant, but the conclusion of the R3 draft is the same as that of the R2 draft. I suggest that they do not assert “Our results indicate…” – I suggest the say instead, “Our results suggest the possibility that exposure ….”

*We have modified the conclusion according to your constructive suggestion, thank you.*

They should also include some details of the significant associations in Table 1 of job stress measures and covaried factors with CACS in the conclusion.

*In Table 1, we present the covariates that we adjust our main results with. The results in Table 1 are unadjusted for age, and not part of our research question so we do not consider it a part of the conclusion.*

In response to my suggestion that they test whether factors they found associated with increased CACS in Table 1 are also associated with job stress measures, they say they have “checked this and none of the adjustment factors was significantly related to high strain jobs, among neither men nor women.” And “However we will not include this in the results section since this is not our research question.” I disagree and recommend they include that these associations were tested and all were nonsignificant.

*We have added a sentence in the results section, first paragraph, according to your recommendation.*

They also say they did not explore associations between components of the metabolic syndrome that are accounting for the association between metabolic syndrome and increased CACS in both men and women because “This study was not an exploratory study…We do not believe it is correct to set up a new research question within the same study when finding out that we have insignificant results.” I also disagree with this argument – indeed, when one has insignificant results, it can be considered appropriate to carry out additional analyses to try to contribute something more than failure to confirm their hypothesis that job stress would be associated with increased CACS.

*We appreciate your interest in which factors affect CACS but we do not consider it appropriate to change our research question and we do not have enough statistical power to do the suggested analyses.*

In response to my concern that it is not clear how doing stratified analyses of job stress associations with CACS in men with MetSyn vs those with no MetSyn addresses the question of mediation of job stress impact on CACS, they say they therefore did the stratified analyses, but say nothing regarding implications of these analyses being nonsignifcant.

*We have added a part in the discussion section, third paragraph, on this topic, thank you for your remark.*
