# Supplementary material for: Psychosocial job exposure and risk of coronary artery calcification
Source: PLoS One. 2021 May 25;16(5):e0252192. doi: 10.1371/journal.pone.0252192 (PMC8148350; doi:10.1371/journal.pone.0252192)
Supplement: Peer review history

# Peer review: Decision Letter 0

**DOI:** 10.1371/journal.pone.0252192.r001

**Authors:** Xianwu Cheng

9 Nov 2020

PONE-D-20-20980

Psychosocial job exposure and risk of coronary artery calcification

PLOS ONE

Dear Dr.  Eriksson

Thank you for submitting your manuscript to PLOS ONE. After careful consideration, we feel that it has merit but does not fully meet PLOS ONE’s publication criteria as it currently stands. Therefore, we invite you to submit a revised version of the manuscript that addresses the points raised during the review process.

Please submit your revised manuscript by December 25, 2020. If you will need more time than this to complete your revisions, please reply to this message or contact the journal office at plosone@plos.org. When you're ready to submit your revision, log on to https://www.editorialmanager.com/pone/ and select the 'Submissions Needing Revision' folder to locate your manuscript file.

Please include the following items when submitting your revised manuscript:

- A rebuttal letter that responds to each point raised by the academic editor and reviewer(s). You should upload this letter as a separate file labeled 'Response to Reviewers'.
- A marked-up copy of your manuscript that highlights changes made to the original version. You should upload this as a separate file labeled 'Revised Manuscript with Track Changes'.
- An unmarked version of your revised paper without tracked changes. You should upload this as a separate file labeled 'Manuscript'.

If you would like to make changes to your financial disclosure, please include your updated statement in your cover letter. Guidelines for resubmitting your figure files are available below the reviewer comments at the end of this letter.

If applicable, we recommend that you deposit your laboratory protocols in protocols.io to enhance the reproducibility of your results. Protocols.io assigns your protocol its own identifier (DOI) so that it can be cited independently in the future. For instructions see: http://journals.plos.org/plosone/s/submission-guidelines#loc-laboratory-protocols

We look forward to receiving your revised manuscript.

Kind regards,

Xianwu Cheng, M.D., Ph.D., FAHA

Academic Editor

PLOS ONE

Journal Requirements:

When submitting your revision, we need you to address these additional requirements.

1. Please ensure that your manuscript meets PLOS ONE's style requirements, including those for file naming. The PLOS ONE style templates can be found at

https://journals.plos.org/plosone/s/file?id=wjVg/PLOSOne_formatting_sample_main_body.pdf and

https://journals.plos.org/plosone/s/file?id=ba62/PLOSOne_formatting_sample_title_authors_affiliations.pdf

2. Please address the following:

- Please ensure you have thoroughly discussed all potential limitations of this study within the Discussion section, including the potential impact of confounding factors.

- Please include additional information regarding the survey or questionnaire used in the study and ensure that you have provided sufficient details that others could replicate the analyses. For instance, if you developed a questionnaire as part of this study and it is not under a copyright more restrictive than CC-BY, please include a copy, in both the original language and English, as Supporting Information.

3.We note that you have indicated that data from this study are available upon request. PLOS only allows data to be available upon request if there are legal or ethical restrictions on sharing data publicly. For information on unacceptable data access restrictions, please see http://journals.plos.org/plosone/s/data-availability#loc-unacceptable-data-access-restrictions.

In your revised cover letter, please address the following prompts:

a) If there are ethical or legal restrictions on sharing a de-identified data set, please explain them in detail (e.g., data contain potentially identifying or sensitive patient information) and who has imposed them (e.g., an ethics committee). Please also provide contact information for a data access committee, ethics committee, or other institutional body to which data requests may be sent.

b) If there are no restrictions, please upload the minimal anonymized data set necessary to replicate your study findings as either Supporting Information files or to a stable, public repository and provide us with the relevant URLs, DOIs, or accession numbers. Please see http://www.bmj.com/content/340/bmj.c181.long for guidelines on how to de-identify and prepare clinical data for publication. For a list of acceptable repositories, please see http://journals.plos.org/plosone/s/data-availability#loc-recommended-repositories.

We will update your Data Availability statement on your behalf to reflect the information you provide.

4. We noted in your submission details that a portion of your manuscript may have been presented or published elsewhere.

[An early draft of the manuscript has been published in paper, but not digitally, in a thesis, the thesis of the corresponding author. It is not possible to upload the whole thesis. I can send you one by mail if you like? ]

Please clarify whether this [conference proceeding or publication] was peer-reviewed and formally published. If this work was previously peer-reviewed and published, in the cover letter please provide the reason that this work does not constitute dual publication and should be included in the current manuscript.

[Note: HTML markup is below. Please do not edit.]

Reviewers' comments:

Reviewer's Responses to Questions

**Comments to the Author**

1. Is the manuscript technically sound, and do the data support the conclusions?

The manuscript must describe a technically sound piece of scientific research with data that supports the conclusions. Experiments must have been conducted rigorously, with appropriate controls, replication, and sample sizes. The conclusions must be drawn appropriately based on the data presented. 

Reviewer #1: Partly

Reviewer #2: Yes

**********

2. Has the statistical analysis been performed appropriately and rigorously? 

Reviewer #1: No

Reviewer #2: No

**********

3. Have the authors made all data underlying the findings in their manuscript fully available?

The PLOS Data policy requires authors to make all data underlying the findings described in their manuscript fully available without restriction, with rare exception (please refer to the Data Availability Statement in the manuscript PDF file). The data should be provided as part of the manuscript or its supporting information, or deposited to a public repository. For example, in addition to summary statistics, the data points behind means, medians and variance measures should be available. If there are restrictions on publicly sharing data—e.g. participant privacy or use of data from a third party—those must be specified.

Reviewer #1: No

Reviewer #2: Yes

**********

4. Is the manuscript presented in an intelligible fashion and written in standard English?

PLOS ONE does not copyedit accepted manuscripts, so the language in submitted articles must be clear, correct, and unambiguous. Any typographical or grammatical errors should be corrected at revision, so please note any specific errors here.

Reviewer #1: Yes

Reviewer #2: Yes

**********

5. Review Comments to the Author

Please use the space provided to explain your answers to the questions above. You may also include additional comments for the author, including concerns about dual publication, research ethics, or publication ethics. (Please upload your review as an attachment if it exceeds 20,000 characters)

Reviewer #1: This ms reports results of analyses of the associations of established measures of stress at work (high job strain, active job and passive job) with coronary artery calcium score (CACS, an established measure of coronary atherosclerosis) in a sample of healthy Swedes on whom they also have measures of the metabolic syndrome (waist circumference, triglycerides, HDL cholesterol, blood pressure and fasting glucose). They report in men non-significant associations of high job strain and active job with higher prevalence ratios (PR) of CACS >100 and in women non-significant association of passive job with higher PR of CACS >100. There are several concerns regarding the statistical analyses the authors used that need to be addressed before a final decision can be reached regarding this ms.

Rather than only doing Cox regression analyses evaluating associations between the job stress measures with PR of CACS 1-99 or CACS > 100 with only age-adjustment or age-adjustment and adjustment for education, smoking, SES area and metabolic syndrome, they need to do more comprehensive analyses that take into account associations of the job stress and CACS measures with the variables they adjust for. In Table 1, for example, men with CACS >100 appear to have higher level of metabolic syndrome (52%) than men with CACs=0 (21%), they also have higher smoking rate (70%) than men with CACS=0(41%). There is no mention in the Results of whether these associations of CACS>100 with increased metabolic syndrome and smoking are statistically significant. It could also be informative if they carried out analyses of the associations between CACs and the components of the metabolic syndrome. That is, are some of the components of the metabolic syndrome – e.g. glucose, triglycerides and BP – accounting for the association of the metabolic syndrome with CACS>100.

They also need to evaluate associations between the job stress measures and all the variables they adjust for in the regression analyses. If, for example, higher job strain is associated with the same factors—i.e., smoking and metabolic syndrome – that appear to be associated with CACS>100, it could be the case that there is a statistically significant indirect pathway from high job strain à metabolic syndrome (or some of its components) and smoking àincreased CACS. They do cite a paper (30) in the Discussion that found job strain associated with increased risk of metabolic syndrome. They also cite a paper in the Introduction (11, Nyborg et al.) that found increased job strain associated with increased diabetes, smoking, physical inactivity and obesity. A paper by Jensen et al. (Circ Cardiovasc Imaging Epub, July 14, 2020) found obesity associated with increased CAC and increased CHD and all cause mortality over a follow-up period.

They could test this pathway using structural equation modeling. If this indirect pathway from job stress indices to increased CACS is found significant, this paper would be making a far greater contribution to our understanding of the impact of increased stress at work on mechanisms involved in the development of coronary artery disease.

There is another measure of stress at work, effort/reward imbalance (ERI), that might be worth evaluating using the same approach (especially as expanded in line with concerns above).

Reviewer #2: The authors provide an interesting and potential important manuscript describing "psychosocial job exposure and risk of coronary artery calcification", The main issues concerning this paper are those concerning the potential associations between psychosocial exposures and coronary calcium.

There are some weak points that need to be addressed by the authors.

Major

1. The author should supplement CACS, the Swedish Demand-Control-Support Questionnaire, the specific evaluation criteria for calcification and working stress。

2. I think the direction chosen in this paper is very good. But the conclusion is bad supported by the data in this paper. First the conclusion say “exposure to a high strain or active job situation could potentially increase the risk of CACS in men”, We don't know if these people are representative of people in social groups who work under different pressures. Second in 384 men, there are 59 people in low strain job, 58 people in active job,111 people in high strain job,156 people in passive job, We can see that most people live in stressful jobs, so it is unscientific to conclude that stressed people have more calcification.

**********

6. PLOS authors have the option to publish the peer review history of their article (what does this mean?). If published, this will include your full peer review and any attached files.

If you choose “no”, your identity will remain anonymous but your review may still be made public.

**Do you want your identity to be public for this peer review?** For information about this choice, including consent withdrawal, please see our Privacy Policy.

Reviewer #1: No

Reviewer #2: No

[NOTE: If reviewer comments were submitted as an attachment file, they will be attached to this email and accessible via the submission site. Please log into your account, locate the manuscript record, and check for the action link "View Attachments". If this link does not appear, there are no attachment files.]

While revising your submission, please upload your figure files to the Preflight Analysis and Conversion Engine (PACE) digital diagnostic tool, https://pacev2.apexcovantage.com/. PACE helps ensure that figures meet PLOS requirements. To use PACE, you must first register as a user. Registration is free. Then, login and navigate to the UPLOAD tab, where you will find detailed instructions on how to use the tool. If you encounter any issues or have any questions when using PACE, please email PLOS at figures@plos.org. Please note that Supporting Information files do not need this step.

# Author comment: Author response to Decision Letter 0

**DOI:** 10.1371/journal.pone.0252192.r002

15 Dec 2020

Response to reviewers

1. Please ensure that your manuscript meets PLOS ONE's style requirements, including those for file naming. The PLOS ONE style templates can be found at

https://journals.plos.org/plosone/s/file?id=wjVg/PLOSOne_formatting_sample_main_body.pdf and

https://journals.plos.org/plosone/s/file?id=ba62/PLOSOne_formatting_sample_title_authors_affiliations.pdf

Thank you for your reminder. The table titles and some of the text in the tables has been changed to bold letters. The affiliations have been adjusted. 

2. Please address the following:

- Please ensure you have thoroughly discussed all potential limitations of this study within the Discussion section, including the potential impact of confounding factors.

Thank you for your comment. We have now further developed the limitation section. Regarding the influence of confounding factors, we had information on smoking, gender, blood lipids and blood pressure which we adjust for and present in the manuscript, before and after adjustment. We lacked information on physical activity which could be considered a confounder, we have added this to the limitation section. 

Please include additional information regarding the survey or questionnaire used in the study and ensure that you have provided sufficient details that others could replicate the analyses. For instance, if you developed a questionnaire as part of this study and it is not under a copyright more restrictive than CC-BY, please include a copy, in both the original language and English, as Supporting Information.

The participants in SCAPIS filled-in a questionnaire covering many different domains, for example smoking and job strain. The questions regarding job strain constitutes an established instrument that has been previously validated in a study by Sanne et al 2003. We will upload the reference with the validation study and where you also can see the questions. 

We note that you have indicated that data from this study are available upon request. PLOS only allows data to be available upon request if there are legal or ethical restrictions on sharing data publicly. For information on unacceptable data access restrictions, please see http://journals.plos.org/plosone/s/data-availability#loc-unacceptable-data-access-restrictions.

In your revised cover letter, please address the following prompts:

a) If there are ethical or legal restrictions on sharing a de-identified data set, please explain them in detail (e.g., data contain potentially identifying or sensitive patient information) and who has imposed them (e.g., an ethics committee). Please also provide contact information for a data access committee, ethics committee, or other institutional body to which data requests may be sent.

b) If there are no restrictions, please upload the minimal anonymized data set necessary to replicate your study findings as either Supporting Information files or to a stable, public repository and provide us with the relevant URLs, DOIs, or accession numbers. Please see http://www.bmj.com/content/340/bmj.c181.long for guidelines on how to de-identify and prepare clinical data for publication. For a list of acceptable repositories, please see http://journals.plos.org/plosone/s/data-availability#loc-recommended-repositories.

We will update your Data Availability statement on your behalf to reflect the information you provide.

Data cannot be shared publicly because information about health and symptoms are regarded as sensitive information, and when sharing such data there has to be an approval from a Swedish Ethical committee (according to Swedish law). However, anonymised data is available with an approval from an ethical review board. For data requests, contact: scapis@scapis.org. The name of the data set is SCAPIS-pilot. We will also add this information to the cover letter.

4. We noted in your submission details that a portion of your manuscript may have been presented or published elsewhere.

[An early draft of the manuscript has been published in paper, but not digitally, in a thesis, the thesis of the corresponding author. It is not possible to upload the whole thesis. I can send you one by mail if you like? ]

Please clarify whether this [conference proceeding or publication] was peer-reviewed and formally published. If this work was previously peer-reviewed and published, in the cover letter please provide the reason that this work does not constitute dual publication and should be included in the current manuscript.

We apologies for not being clear on this point. The thesis is the first author’s PhD-thesis/doctoral dissertation and was only presented orally during the viva, i.e. the "dissertation defence” but it has not been peer-reviewed nor formally published, only printed in paper in a limited amount as part of the PhD-thesis. 

Reviewer #1: This ms reports results of analyses of the associations of established measures of stress at work (high job strain, active job and passive job) with coronary artery calcium score (CACS, an established measure of coronary atherosclerosis) in a sample of healthy Swedes on whom they also have measures of the metabolic syndrome (waist circumference, triglycerides, HDL cholesterol, blood pressure and fasting glucose). They report in men non-significant associations of high job strain and active job with higher prevalence ratios (PR) of CACS >100 and in women non-significant association of passive job with higher PR of CACS >100. There are several concerns regarding the statistical analyses the authors used that need to be addressed before a final decision can be reached regarding this ms.

Thank you for the summary. Please let us know if the concerns regarding the statistical analyses are about the analyses done or those not done. 

Rather than only doing Cox regression analyses evaluating associations between the job stress measures with PR of CACS 1-99 or CACS > 100 with only age-adjustment or age-adjustment and adjustment for education, smoking, SES area and metabolic syndrome, they need to do more comprehensive analyses that take into account associations of the job stress and CACS measures with the variables they adjust for. In Table 1, for example, men with CACS >100 appear to have higher level of metabolic syndrome (52%) than men with CACs=0 (21%), they also have higher smoking rate (70%) than men with CACS=0(41%). There is no mention in the Results of whether these associations of CACS>100 with increased metabolic syndrome and smoking are statistically significant. It could also be informative if they carried out analyses of the associations between CACs and the components of the metabolic syndrome. That is, are some of the components of the metabolic syndrome – e.g. glucose, triglycerides and BP – accounting for the association of the metabolic syndrome with CACS>100.

Thank you for your comments. Yes, we always want to know more, however the aim of the study was to analyze if exposure to job strain was associated with development of coronary artery calcium, CAC, not what factors that causes CAC. That is why we do not present these associations in the results, although in Methods we have presented whether the associations are significant: ”a second model with adjustment for age, smoking, education, socioeconomic area and metabolic syndrome (all p<0.05 when adding one by one to the first model for CACS >100 and all but socioeconomic area p<0.05 for CACS 1-99).” The study is also too small to go into details of the components of the metabolic syndrome. We hope to do that in the larger study. 

They also need to evaluate associations between the job stress measures and all the variables they adjust for in the regression analyses. If, for example, higher job strain is associated with the same factors—i.e., smoking and metabolic syndrome – that appear to be associated with CACS>100, it could be the case that there is a statistically significant indirect pathway from high job strain à metabolic syndrome (or some of its components) and smoking àincreased CACS. They do cite a paper (30) in the Discussion that found job strain associated with increased risk of metabolic syndrome. They also cite a paper in the Introduction (11, Nyborg et al.) that found increased job strain associated with increased diabetes, smoking, physical inactivity and obesity. A paper by Jensen et al. (Circ Cardiovasc Imaging Epub, July 14, 2020) found obesity associated with increased CAC and increased CHD and all cause mortality over a follow-up period.

They could test this pathway using structural equation modeling. If this indirect pathway from job stress indices to increased CACS is found significant, this paper would be making a far greater contribution to our understanding of the impact of increased stress at work on mechanisms involved in the development of coronary artery disease.

Thank you for your comments. The aim of the study was to analyze if exposure to job demand-control was associated to development of coronary artery calcium. The standard procedure in this area of research is to establish association between work exposure and an outcome, and if that association remains when entering known covariates. Although we would think it would produce interesting results, illustrating possible pathways between stressful job exposure and health, in this cross-sectional data it is not possible since mediator analyses require the temporal aspect of x�M � y. The main SCAPIS cohort, from which the data used in this paper is the pilot data, will be a longitudinal study, where we hope to perform the suggested analyses in the future. We are currently in the process of initiating another study on the core cohort material where we aim to establish interactive effects from job exposure are markers for socioeconomic status, since it’s a much larger material and will take these insightful comments into consideration. 

We have not presented data about correlations between the used covariates, we will add that. Gender was the one with highest correlation, r=-0.33 (both with Pearson and Spearman), and we present the results for both genders separately. All other covariates, outcome and four kinds of job stress was less correlated than r=0.3 to each other. We have made stratified analyses for metabolic syndrome, ever-smoking and socioeconomic area, respectively (not shown), among men and CACS>100 without finding any major changes between strata or in results. For women, there are too few cases.

There is another measure of stress at work, effort/reward imbalance (ERI), that might be worth evaluating using the same approach (especially as expanded in line with concerns above).

Thank you for your suggestion, unfortunately we do not have data on exposure to effort reward imbalance, the participants have not answered questions concerning effort/reward.

Reviewer #2: The authors provide an interesting and potential important manuscript describing "psychosocial job exposure and risk of coronary artery calcification", The main issues concerning this paper are those concerning the potential associations between psychosocial exposures and coronary calcium. 

There are some weak points that need to be addressed by the authors.

Major

1. The author should supplement CACS, the Swedish Demand-Control-Support Questionnaire, the specific evaluation criteria for calcification and working stress. 

The amount of coronary artery calcium is frequently quantified through the Agatston scoring method where calcium deposits areas are multiplied by a density factor estimated through a computed tomography investigation. The Agatston scoring method is described in reference 23, which we will supplement (Agatston AS, Janowitz WR, Hildner FJ, Zusmer NR, Viamonte M, Jr., Detrano R. Quantification of coronary artery calcium using ultrafast computed tomography. J Am Coll Cardiol. 1990;15:827-32). We will also supplement reference 26 where one can see the questions that we have used (Sanne B, Torp S, Mykletun A, Dahl AA. The Swedish Demand-Control-Support Questionnaire (DCSQ): factor structure, item analyses, and internal consistency in a large population. Scand J Public Health. 2005;33:166-74).

2. I think the direction chosen in this paper is very good. But the conclusion is bad supported by the data in this paper. First the conclusion say “exposure to a high strain or active job situation could potentially increase the risk of CACS in men”, We don't know if these people are representative of people in social groups who work under different pressures. Second in 384 men, there are 59 people in low strain job, 58 people in active job,111 people in high strain job,156 people in passive job, We can see that most people live in stressful jobs, so it is unscientific to conclude that stressed people have more calcification.

Thank you for your comment. According to the model theory, developed by Karasek and Theorell adverse health effects from work exposure is determined by the volume of work tasks (demand) and positive buffering effects from work control (Karasek R, Theorell T. Healthy work; stress, productivity and the reconstruction of working life. New York, N.Y: Basic Books; 1990). Thus the categorization of participants into: 1. Low strain job =low demands/high control 2. Active job=high demands/high control 3. Passive job=low demands/low control 4. High strain job=high demands/low control, creates a categorization of 4 different work situations. High demand combined with low control is regarded as the most detrimental work type, which has been established in a wide range of studies. Traditionally “active” work is conceived as intense but also stimulating and not hazardous as the workers influence over the work situation may lower adverse effects from high work volumes. Passive job, low demands-low control, has been conceived as either positive or negative in the first decades of research with this model, but in recent years there has been evidence that this more monotonous work situation may for example spill over to the leisure time, as workers with passive jobs tend to have less physical activity in their leisure time. Low strain (low demand-high control) is generally assigned as a reference and a preferred work situation as it entails low stress from high amounts of work tasks, but also high freedom within one’s job.

Attachment Submitted filename: Response to reviewers_coronarycalciumdec20_helenaeriksson.docx Click here for additional data file.

# Peer review: Decision Letter 1

**DOI:** 10.1371/journal.pone.0252192.r003

**Authors:** Xianwu Cheng

4 Jan 2021

PONE-D-20-20980R1

Psychosocial job exposure and risk of coronary artery calcification

PLOS ONE

Dear Dr.  Eriksson

Thank you for submitting your manuscript to PLOS ONE. After careful consideration, we feel that it has merit but does not fully meet PLOS ONE’s publication criteria as it currently stands. Therefore, we invite you to submit a revised version of the manuscript that addresses the points raised during the review process.

Please submit your revised manuscript by %March 2, 2021. If you will need more time than this to complete your revisions, please reply to this message or contact the journal office at plosone@plos.org. When you're ready to submit your revision, log on to https://www.editorialmanager.com/pone/ and select the 'Submissions Needing Revision' folder to locate your manuscript file.

Please include the following items when submitting your revised manuscript:

- A rebuttal letter that responds to each point raised by the academic editor and reviewer(s). You should upload this letter as a separate file labeled 'Response to Reviewers'.
- A marked-up copy of your manuscript that highlights changes made to the original version. You should upload this as a separate file labeled 'Revised Manuscript with Track Changes'.
- An unmarked version of your revised paper without tracked changes. You should upload this as a separate file labeled 'Manuscript'.

If you would like to make changes to your financial disclosure, please include your updated statement in your cover letter. Guidelines for resubmitting your figure files are available below the reviewer comments at the end of this letter.

If applicable, we recommend that you deposit your laboratory protocols in protocols.io to enhance the reproducibility of your results. Protocols.io assigns your protocol its own identifier (DOI) so that it can be cited independently in the future. For instructions see: http://journals.plos.org/plosone/s/submission-guidelines#loc-laboratory-protocols

We look forward to receiving your revised manuscript.

Kind regards,

Xianwu Cheng, M.D., Ph.D., FAHA

Academic Editor

PLOS ONE

Additional Editor Comments (if provided):

One original reviewer has still concerned that the authors did not satisfactory addressed most of original comments. Thus, this is last chance to revise your manuscript for pulication in PONE.

[Note: HTML markup is below. Please do not edit.]

Reviewers' comments:

Reviewer's Responses to Questions

**Comments to the Author**

1. If the authors have adequately addressed your comments raised in a previous round of review and you feel that this manuscript is now acceptable for publication, you may indicate that here to bypass the “Comments to the Author” section, enter your conflict of interest statement in the “Confidential to Editor” section, and submit your "Accept" recommendation.

Reviewer #1: (No Response)

Reviewer #2: All comments have been addressed

**********

2. Is the manuscript technically sound, and do the data support the conclusions?

The manuscript must describe a technically sound piece of scientific research with data that supports the conclusions. Experiments must have been conducted rigorously, with appropriate controls, replication, and sample sizes. The conclusions must be drawn appropriately based on the data presented. 

Reviewer #1: Partly

Reviewer #2: Yes

**********

3. Has the statistical analysis been performed appropriately and rigorously? 

Reviewer #1: No

Reviewer #2: Yes

**********

4. Have the authors made all data underlying the findings in their manuscript fully available?

The PLOS Data policy requires authors to make all data underlying the findings described in their manuscript fully available without restriction, with rare exception (please refer to the Data Availability Statement in the manuscript PDF file). The data should be provided as part of the manuscript or its supporting information, or deposited to a public repository. For example, in addition to summary statistics, the data points behind means, medians and variance measures should be available. If there are restrictions on publicly sharing data—e.g. participant privacy or use of data from a third party—those must be specified.

Reviewer #1: Yes

Reviewer #2: Yes

**********

5. Is the manuscript presented in an intelligible fashion and written in standard English?

PLOS ONE does not copyedit accepted manuscripts, so the language in submitted articles must be clear, correct, and unambiguous. Any typographical or grammatical errors should be corrected at revision, so please note any specific errors here.

Reviewer #1: Yes

Reviewer #2: Yes

**********

6. Review Comments to the Author

Please use the space provided to explain your answers to the questions above. You may also include additional comments for the author, including concerns about dual publication, research ethics, or publication ethics. (Please upload your review as an attachment if it exceeds 20,000 characters)

Reviewer #1: The authors have not responded adequately to my concerns regarding the original version of this ms. With a sample of 384 men and 393 women, there is ample power for them to conduct and report results of exploratory tests of associations between the variables adjusted for in the analyses of associations between job stress measures and CACS and the job stress measures and CACS levels themselves. They do note in Methods (lines 153-156) that the covariates (age, smoking, education, socioeconomic area and metabolic syndrome) were “all P< 0.05 when adding one by one to the first model for CACS > 100..” If I interpret this correctly, this means that all these covariates were significantly associated significantly with severe CACS > 100. There is no reason they should not report these associations in a table – or indicate in Table 1 that the 52% of men with CACS >100 is significantly different from the 21% of men with metabolic syndrome in those with CACS of 0, with similar indicates for the other covariates.

In addition to these presumed associations of the covaried risk factors with CACS, attention also needs to be given to their associations with the job stress measures. If, as I noted in my review of the original ms, there is a significant association of high job strain with high levels of metabolic syndrome, it would suggest the possibility, even if they do not provide a direct test of metabolic syndrome as a mediator, that the increased CACS in men with high job strain may be the result of their higher level of metabolic syndrome. Given that prior research (ref 30) has found an association between high job strain and metabolic syndrome, there is every reason to expect they will be able to replicate that finding in the current study. I don’t disagree with their point that using SEM to assess possible pathways is now considered to require the temporal aspect to be conclusive with respect to causation. It might still be informative, however, even with the current cross-sectional data, to perform SEM pathway analysis, as an exploratory way of seeing if there might be a pathway from job strain to higher CACS via metabolic syndrome. Even though it could not be considered definitive (which the authors would note in a revised ms), if such an SEM path analysis did identify a statistically significant indirect pathway from high job strain to high metabolic syndrome (or other covariates or metabolic syndrome components per below) and from there to high CACS, it would be encouraging that, despite their failure to find a significant association between job strain and CACS, there is reason to continue to pursue ongoing research (including longitudinal) to evaluate job stress measures as risk factors for CACS (and CVD events). Moreover, finding such a significant indirect pathway could also help identify intervention targets in men in high strain jobs – e.g., if there’s a significant indirect pathway from job strain via smoking to CACS, that would suggest stopping smoking as an important preventive approach in men in high strain jobs.

Speaking of exploratory analyses, there is no reason, given a total sample of 777 men and women, for them to assert that “the study is too small to go into details of the components of the metabolic syndrome.” If the metabolic syndrome itself (and other covariates) are significantly associated with CACS (and job stress measures?), it should be possible to determine whether the association of metabolic syndrome itself with CADS is due to one or more specific components of the metabolic syndrome.

They conclude that “Our results indicate that exposure for high strain job or active job could increase the risk of CAC in men, but in women, it could rather be exposure for passive job that increases the risk. However, there was a lack of power in the study.” Given that the associations of job stress measures with CACS in men and women were not statistically significant, I believe it would behoove the authors to perform exploratory analyses as outlined in my comments above to take maximum advantage of the extensive data they have on this sample of 384 men and 393 women.

Reviewer #2: Dear author

Thank you very much for providing the supplement CACS and explain the categorization of participants. This paper may highlight the psychological factor on vascular calcification, which may notice people care for the work stress.

**********

7. PLOS authors have the option to publish the peer review history of their article (what does this mean?). If published, this will include your full peer review and any attached files.

If you choose “no”, your identity will remain anonymous but your review may still be made public.

**Do you want your identity to be public for this peer review?** For information about this choice, including consent withdrawal, please see our Privacy Policy.

Reviewer #1: No

Reviewer #2: No

[NOTE: If reviewer comments were submitted as an attachment file, they will be attached to this email and accessible via the submission site. Please log into your account, locate the manuscript record, and check for the action link "View Attachments". If this link does not appear, there are no attachment files.]

While revising your submission, please upload your figure files to the Preflight Analysis and Conversion Engine (PACE) digital diagnostic tool, https://pacev2.apexcovantage.com/. PACE helps ensure that figures meet PLOS requirements. To use PACE, you must first register as a user. Registration is free. Then, login and navigate to the UPLOAD tab, where you will find detailed instructions on how to use the tool. If you encounter any issues or have any questions when using PACE, please email PLOS at figures@plos.org. Please note that Supporting Information files do not need this step.

Attachment Submitted filename: Response-PONE-D-20-20980_R1.doc Click here for additional data file.

# Author comment: Author response to Decision Letter 1

**DOI:** 10.1371/journal.pone.0252192.r004

19 Jan 2021

In the Abstract we have made some changes to make it clearer. Also, in Introduction first paragraph, we have added some text regarding the job strain model to try to make it more clear for the reader. 

Reviewer #1: The authors have not responded adequately to my concerns regarding the original version of this ms. With a sample of 384 men and 393 women, there is ample power for them to conduct and report results of exploratory tests of associations between the variables adjusted for in the analyses of associations between job stress measures and CACS and the job stress measures and CACS levels themselves.

Thank you for your recommendations on further analyses. We regret if our methodology has not been clear enough to illustrate that in this study the participants with established CAC are divided into four exposure groups, depending on their reporting of high or low job demand or job control, hence, there are sometimes few cases in each exposure group. If one further divides the participants according to for example presence of the metabolic syndrome there will be even less cases in each exposure group. Please see the table below where we have tried to clarify this. As the amount of cases are few, the statistical power would be too low for further analysis of potential associations.

Cases with CACS>100 among men by job type and metabolic syndrome. Each of the other job types are compared with low strain job in the analyses. There are 4-5 cases in the reference group. (Please see a correct version of the mentioned table above in response to reviewers letter, it was not possible to include it here in this text box) 

Low strain job High strain job Active job Passive job Total

No metabolic syndrome 5 11 9 12 37

Metabolic syndrome 4 18 7 11 40

Total 9 29 16 23 77

They do note in Methods (lines 153-156) that the covariates (age, smoking, education, socioeconomic area and metabolic syndrome) were “all P< 0.05 when adding one by one to the first model for CACS > 100..” If I interpret this correctly, this means that all these covariates were significantly associated significantly with severe CACS > 100. There is no reason they should not report these associations in a table – or indicate in Table 1 that the 52% of men with CACS >100 is significantly different from the 21% of men with metabolic syndrome in those with CACS of 0, with similar indicates for the other covariates.

Thank you, we have now marked these significant differences in table 1, we have also added explanatory text to table 1 and text in Methods/Statistical analyses, third sentence. 

In addition to these presumed associations of the covaried risk factors with CACS, attention also needs to be given to their associations with the job stress measures. If, as I noted in my review of the original ms, there is a significant association of high job strain with high levels of metabolic syndrome, it would suggest the possibility, even if they do not provide a direct test of metabolic syndrome as a mediator, that the increased CACS in men with high job strain may be the result of their higher level of metabolic syndrome. 

For several reasons, please also see our first answer in this letter and further down, we cannot do the suggested analyses, but we can present a stratified analysis regarding metabolic syndrome which hopefully provides the reviewer with some answers. In Results, last section, page 10 we have now presented the results for stratification of men in to those with the metabolic syndrome and those without it, the risk of CACS>100 among men with high strain job but no metabolic syndrome was PR 1.42 (95% CI 0.58-3.44) and among men with high strain job and metabolic syndrome was PR 1.62 (95% CI 0.80-3.30) (both adjusted for the significant covariates age and ever-smoking). 

Given that prior research (ref 30) has found an association between high job strain and metabolic syndrome, there is every reason to expect they will be able to replicate that finding in the current study. I don’t disagree with their point that using SEM to assess possible pathways is now considered to require the temporal aspect to be conclusive with respect to causation. It might still be informative, however, even with the current cross-sectional data, to perform SEM pathway analysis, as an exploratory way of seeing if there might be a pathway from job strain to higher CACS via metabolic syndrome. Even though it could not be considered definitive (which the authors would note in a revised ms), if such an SEM path analysis did identify a statistically significant indirect pathway from high job strain to high metabolic syndrome (or other covariates or metabolic syndrome components per below) and from there to high CACS, it would be encouraging that, despite their failure to find a significant association between job strain and CACS, there is reason to continue to pursue ongoing research (including longitudinal) to evaluate job stress measures as risk factors for CACS (and CVD events). Moreover, finding such a significant indirect pathway could also help identify intervention targets in men in high strain jobs – e.g., if there’s a significant indirect pathway from job strain via smoking to CACS, that would suggest stopping smoking as an important preventive approach in men in high strain jobs.

Speaking of exploratory analyses, there is no reason, given a total sample of 777 men and women, for them to assert that “the study is too small to go into details of the components of the metabolic syndrome.” If the metabolic syndrome itself (and other covariates) are significantly associated with CACS (and job stress measures?), it should be possible to determine whether the association of metabolic syndrome itself with CADS is due to one or more specific components of the metabolic syndrome.

Thank you for your comments and your interest in the complex possible pathways between psychosocial stressors and cardiovascular outcomes. We provided our limitations in terms of lacking statistical power under the first paragraph of your revision requests. 

Regarding adding analyses of metabolic syndrome: There is throughout the literature established relationships between adverse psychosocial job exposure and coronary heart disease, such as myocardial infarction. However, the intermediary pathways are not known. The aim of this study was to study associations between exposure to work related stress and CAC, which is a plausible pathway in the disease chain between job stressors and CHD. We feel that since the focused outcome is already an intermediary variable, examining yet another intermediary variable and its relationship to CAC, would make the study less focused, especially since this is a cross-sectional study and causality cannot be established between CAC and metabolic syndrome. Furthermore, in occupational medicine it is common to analyse the job exposure, and the strength of that association when adjusting for several covariates, without reporting the associations of the covariates, as they are not the focus or the variables of interest. 

They conclude that “Our results indicate that exposure for high strain job or active job could increase the risk of CAC in men, but in women, it could rather be exposure for passive job that increases the risk. However, there was a lack of power in the study.” Given that the associations of job stress measures with CACS in men and women were not statistically significant, I believe it would behoove the authors to perform exploratory analyses as outlined in my comments above to take maximum advantage of the extensive data they have on this sample of 384 men and 393 women.

Please see our previous answers above regarding power. The power is based on the amount of cases, who when divided into the four different psychosocial job categories, are few in several cells. 

The SCAPIS-pilot was performed to check all details in the large SCAPIS protocol but also to have data to predict selection bias from participation rates in different socioeconomic geographical areas (Björk et al. Scand J Public Health 2017;45(Suppl 17):45-49). In order to do that low and high socioeconomic areas were studied. We believed it was interesting to study job strain within a span of different job experiences as they came from both low and high socioeconomic areas, even with a limited population.

Reviewer #2: Dear author

Thank you very much for providing the supplement CACS and explain the categorization of participants. This paper may highlight the psychological factor on vascular calcification, which may notice people care for the work stress.

Thank you for feedback!

________________________________________

7. PLOS authors have the option to publish the peer review history of their article (what does this mean?). If published, this will include your full peer review and any attached files.

If you choose “no”, your identity will remain anonymous but your review may still be made public.

Do you want your identity to be public for this peer review? For information about this choice, including consent withdrawal, please see our Privacy Policy.

Reviewer #1: No

Attachment Submitted filename: resprevjan19herikssoncacsubm.docx Click here for additional data file.

# Peer review: Decision Letter 2

**DOI:** 10.1371/journal.pone.0252192.r005

**Authors:** Xianwu Cheng

9 Feb 2021

PONE-D-20-20980R2

Psychosocial job exposure and risk of coronary artery calcification

PLOS ONE

Dear Dr Eriksson

Thank you for submitting your manuscript to PLOS ONE. After careful consideration, we feel that it has merit but does not fully meet PLOS ONE’s publication criteria as it currently stands. Therefore, we invite you to submit a revised version of the manuscript that addresses the points raised during the review process.

Please submit your revised manuscript by March30, 2021. If you will need more time than this to complete your revisions, please reply to this message or contact the journal office at plosone@plos.org. When you're ready to submit your revision, log on to https://www.editorialmanager.com/pone/ and select the 'Submissions Needing Revision' folder to locate your manuscript file.

Please include the following items when submitting your revised manuscript:

- A rebuttal letter that responds to each point raised by the academic editor and reviewer(s). You should upload this letter as a separate file labeled 'Response to Reviewers'.
- A marked-up copy of your manuscript that highlights changes made to the original version. You should upload this as a separate file labeled 'Revised Manuscript with Track Changes'.
- An unmarked version of your revised paper without tracked changes. You should upload this as a separate file labeled 'Manuscript'.

If you would like to make changes to your financial disclosure, please include your updated statement in your cover letter. Guidelines for resubmitting your figure files are available below the reviewer comments at the end of this letter.

If applicable, we recommend that you deposit your laboratory protocols in protocols.io to enhance the reproducibility of your results. Protocols.io assigns your protocol its own identifier (DOI) so that it can be cited independently in the future. For instructions see: http://journals.plos.org/plosone/s/submission-guidelines#loc-laboratory-protocols

We look forward to receiving your revised manuscript.

Kind regards,

Xianwu Cheng, M.D., Ph.D., FAHA

Academic Editor

PLOS ONE

[Note: HTML markup is below. Please do not edit.]

Reviewers' comments:

Reviewer's Responses to Questions

**Comments to the Author**

1. If the authors have adequately addressed your comments raised in a previous round of review and you feel that this manuscript is now acceptable for publication, you may indicate that here to bypass the “Comments to the Author” section, enter your conflict of interest statement in the “Confidential to Editor” section, and submit your "Accept" recommendation.

Reviewer #1: (No Response)

Reviewer #2: All comments have been addressed

**********

2. Is the manuscript technically sound, and do the data support the conclusions?

The manuscript must describe a technically sound piece of scientific research with data that supports the conclusions. Experiments must have been conducted rigorously, with appropriate controls, replication, and sample sizes. The conclusions must be drawn appropriately based on the data presented. 

Reviewer #1: Partly

Reviewer #2: Yes

**********

3. Has the statistical analysis been performed appropriately and rigorously? 

Reviewer #1: No

Reviewer #2: Yes

**********

4. Have the authors made all data underlying the findings in their manuscript fully available?

The PLOS Data policy requires authors to make all data underlying the findings described in their manuscript fully available without restriction, with rare exception (please refer to the Data Availability Statement in the manuscript PDF file). The data should be provided as part of the manuscript or its supporting information, or deposited to a public repository. For example, in addition to summary statistics, the data points behind means, medians and variance measures should be available. If there are restrictions on publicly sharing data—e.g. participant privacy or use of data from a third party—those must be specified.

Reviewer #1: No

Reviewer #2: Yes

**********

5. Is the manuscript presented in an intelligible fashion and written in standard English?

PLOS ONE does not copyedit accepted manuscripts, so the language in submitted articles must be clear, correct, and unambiguous. Any typographical or grammatical errors should be corrected at revision, so please note any specific errors here.

Reviewer #1: Yes

Reviewer #2: Yes

**********

6. Review Comments to the Author

Please use the space provided to explain your answers to the questions above. You may also include additional comments for the author, including concerns about dual publication, research ethics, or publication ethics. (Please upload your review as an attachment if it exceeds 20,000 characters)

Reviewer #1: The authors have responded to my request to include indication of significant differences comparing CACS 1-99 and >100 groups with the CACs 0 group with respect to job stress measures, ever smoker, SES area, and Metabolic Syndrome measures in Table 1. And it is interesting to observe that in men only ever smoker and MetSyn are higher in CACS 1-99 and CACS >100 than CACS 0, and in women only No University and SES area are higher in CACS >100 and MetSyn is higher in CACS 1-99, but not CACS >100 – likely due to small N (7) in that group. They need to include these statistically significant differences in the Results section.

Despite the fact that none of the tests of associations between job stress and other measures with CACS were significant in either men or women, they still conclude that, despite “a lack of power in the study,” “Our results indicate that exposure for high strain job or active job could increase the risk of CAC in men, but in women, it could rather be exposure for passive job that increases the risk.” Given this lack of significant effects for the job stress measures, a strong case can be made that they should undertake exploratory analyses in this sample of 777 healthy middle-aged men and women to evaluate associations between job stress measures and other factors they find associated with increased CACS – i.e., ever smoker and MetSyn in men and No University, SES area and MetSyn in women – to determine whether these other factors that are associated with CACS and, therefore, at least potential mediators of effects of job stress on CACS, are increased in men and women with high job stress. If these potential mediators are significantly increased in men and women with high levels of job stress measures, it would at least enable the authors to suggest – even if they do not perform SEM path analyses, that these factors (especially ever smoked and MetSyn) could be mediators of associations between job stress and CACS.

A similar argument can be made for them to perform exploratory analyses to determine whether it is possible to identify specific components of the MetSyn that are accounting for the association between MetSyn and increased CACS in both men and women, and, if so, whether these MetSyn components are also elevated in men and/or women with high levels of job stress.

It is not clear how doing stratified analyses of job stress associations with CACS in men with MetSyn vs men with no MetSyn – all nonsignificant – addresses the question of mediation of job stress impact on CACS.

Reviewer #2: Dear author

The paper entitled with Psychosocial job exposure and risk of coronary artery calcification show an interesting study to identify psychological stress on CVD disease, the detail mechanism for calcification need to further explore, but this phenomenon need our of us to notice.

Thank you very much to made a meaningful research.

**********

7. PLOS authors have the option to publish the peer review history of their article (what does this mean?). If published, this will include your full peer review and any attached files.

If you choose “no”, your identity will remain anonymous but your review may still be made public.

**Do you want your identity to be public for this peer review?** For information about this choice, including consent withdrawal, please see our Privacy Policy.

Reviewer #1: No

Reviewer #2: No

[NOTE: If reviewer comments were submitted as an attachment file, they will be attached to this email and accessible via the submission site. Please log into your account, locate the manuscript record, and check for the action link "View Attachments". If this link does not appear, there are no attachment files.]

While revising your submission, please upload your figure files to the Preflight Analysis and Conversion Engine (PACE) digital diagnostic tool, https://pacev2.apexcovantage.com/. PACE helps ensure that figures meet PLOS requirements. To use PACE, you must first register as a user. Registration is free. Then, login and navigate to the UPLOAD tab, where you will find detailed instructions on how to use the tool. If you encounter any issues or have any questions when using PACE, please email PLOS at figures@plos.org. Please note that Supporting Information files do not need this step.

# Author comment: Author response to Decision Letter 2

**DOI:** 10.1371/journal.pone.0252192.r006

3 Mar 2021

Response to reviewers

3rd of March 2021

We have marked all of our changes in yellow in the manuscript. 

Reviewer #1: The authors have responded to my request to include indication of significant differences comparing CACS 1-99 and >100 groups with the CACs 0 group with respect to job stress measures, ever smoker, SES area, and Metabolic Syndrome measures in Table 1. And it is interesting to observe that in men only ever smoker and MetSyn are higher in CACS 1-99 and CACS >100 than CACS 0, and in women only No University and SES area are higher in CACS >100 and MetSyn is higher in CACS 1-99, but not CACS >100 – likely due to small N (7) in that group. They need to include these statistically significant differences in the Results section.

We have now included this in the results section, first paragraph. We have, however, refrained to add further suggested analyses as this is not a part of our study aim, which focus on work exposure and health. 

Despite the fact that none of the tests of associations between job stress and other measures with CACS were significant in either men or women, they still conclude that, despite “a lack of power in the study,” “Our results indicate that exposure for high strain job or active job could increase the risk of CAC in men, but in women, it could rather be exposure for passive job that increases the risk.”

Thank you for your comment, we have moderated the conclusion

Given this lack of significant effects for the job stress measures, a strong case can be made that they should undertake exploratory analyses in this sample of 777 healthy middle-aged men and women to evaluate associations between job stress measures and other factors they find associated with increased CACS – i.e., ever smoker and MetSyn in men and No University, SES area and MetSyn in women – to determine whether these other factors that are associated with CACS and, therefore, at least potential mediators of effects of job stress on CACS, are increased in men and women with high job stress. If these potential mediators are significantly increased in men and women with high levels of job stress measures, it would at least enable the authors to suggest – even if they do not perform SEM path analyses, that these factors (especially ever smoked and MetSyn) could be mediators of associations between job stress and CACS.

We have now checked this and none of the adjustment factors was significantly related to high strain jobs, among neither men nor women. However, we will not include this in the results section, since this is not our research question. 

A similar argument can be made for them to perform exploratory analyses to determine whether it is possible to identify specific components of the MetSyn that are accounting for the association between MetSyn and increased CACS in both men and women, and, if so, whether these MetSyn components are also elevated in men and/or women with high levels of job stress.

This study was not an exploratory study and our research question was to to examine associations between psychosocial job exposures and presence of coronary artery calcium.

We do not believe that it is correct to set up a new research question within the same study when finding out that we have insignificant results. Also, as previously said, we do not have enough statistical power to do the suggested analyses. 

It is not clear how doing stratified analyses of job stress associations with CACS in men with MetSyn vs men with no MetSyn – all nonsignificant – addresses the question of mediation of job stress impact on CACS.

Thank you for your comment. We agree that the metabolic syndrome can be a factor that partly explains the development of CAC, that is why we also have stratified for the metabolic syndrome not only adjusted for it. The stratified analyses may show that it is not only subjects with the metabolic syndrome that seems to be at higher risk of CAC in high strain jobs.

Reviewer #2: Dear author

The paper entitled with Psychosocial job exposure and risk of coronary artery calcification show an interesting study to identify psychological stress on CVD disease, the detail mechanism for calcification need to further explore, but this phenomenon need our of us to notice.

Thank you very much to made a meaningful research.

We are very thankful for your feedback

Attachment Submitted filename: resprev3mars21cacherikssonsubmit.docx Click here for additional data file.

# Peer review: Decision Letter 3

**DOI:** 10.1371/journal.pone.0252192.r007

**Authors:** Xianwu Cheng

24 Mar 2021

PONE-D-20-20980R3

Psychosocial job exposure and risk of coronary artery calcification

PLOS ONE

Dear Dr. Eriksson

Thank you for submitting your manuscript to PLOS ONE. After careful consideration, we feel that it has merit but does not fully meet PLOS ONE’s publication criteria as it currently stands. Therefore, we invite you to submit a revised version of the manuscript that addresses the points raised during the review process.

Please submit your revised manuscript by April 30, 2021. If you will need more time than this to complete your revisions, please reply to this message or contact the journal office at plosone@plos.org. When you're ready to submit your revision, log on to https://www.editorialmanager.com/pone/ and select the 'Submissions Needing Revision' folder to locate your manuscript file.

Please include the following items when submitting your revised manuscript:

- A rebuttal letter that responds to each point raised by the academic editor and reviewer(s). You should upload this letter as a separate file labeled 'Response to Reviewers'.
- A marked-up copy of your manuscript that highlights changes made to the original version. You should upload this as a separate file labeled 'Revised Manuscript with Track Changes'.
- An unmarked version of your revised paper without tracked changes. You should upload this as a separate file labeled 'Manuscript'.

If you would like to make changes to your financial disclosure, please include your updated statement in your cover letter. Guidelines for resubmitting your figure files are available below the reviewer comments at the end of this letter.

If applicable, we recommend that you deposit your laboratory protocols in protocols.io to enhance the reproducibility of your results. Protocols.io assigns your protocol its own identifier (DOI) so that it can be cited independently in the future. For instructions see: http://journals.plos.org/plosone/s/submission-guidelines#loc-laboratory-protocols

We look forward to receiving your revised manuscript.

Kind regards,

Xianwu Cheng, M.D., Ph.D., FAHA

Academic Editor

PLOS ONE

Journal Requirements:

Please review your reference list to ensure that it is complete and correct. If you have cited papers that have been retracted, please include the rationale for doing so in the manuscript text, or remove these references and replace them with relevant current references. Any changes to the reference list should be mentioned in the rebuttal letter that accompanies your revised manuscript. If you need to cite a retracted article, indicate the article’s retracted status in the References list and also include a citation and full reference for the retraction notice.

[Note: HTML markup is below. Please do not edit.]

Reviewers' comments:

Reviewer's Responses to Questions

**Comments to the Author**

1. If the authors have adequately addressed your comments raised in a previous round of review and you feel that this manuscript is now acceptable for publication, you may indicate that here to bypass the “Comments to the Author” section, enter your conflict of interest statement in the “Confidential to Editor” section, and submit your "Accept" recommendation.

Reviewer #1: (No Response)

**********

2. Is the manuscript technically sound, and do the data support the conclusions?

The manuscript must describe a technically sound piece of scientific research with data that supports the conclusions. Experiments must have been conducted rigorously, with appropriate controls, replication, and sample sizes. The conclusions must be drawn appropriately based on the data presented. 

Reviewer #1: Partly

**********

3. Has the statistical analysis been performed appropriately and rigorously? 

Reviewer #1: Yes

**********

4. Have the authors made all data underlying the findings in their manuscript fully available?

The PLOS Data policy requires authors to make all data underlying the findings described in their manuscript fully available without restriction, with rare exception (please refer to the Data Availability Statement in the manuscript PDF file). The data should be provided as part of the manuscript or its supporting information, or deposited to a public repository. For example, in addition to summary statistics, the data points behind means, medians and variance measures should be available. If there are restrictions on publicly sharing data—e.g. participant privacy or use of data from a third party—those must be specified.

Reviewer #1: No

**********

5. Is the manuscript presented in an intelligible fashion and written in standard English?

PLOS ONE does not copyedit accepted manuscripts, so the language in submitted articles must be clear, correct, and unambiguous. Any typographical or grammatical errors should be corrected at revision, so please note any specific errors here.

Reviewer #1: Yes

**********

6. Review Comments to the Author

Please use the space provided to explain your answers to the questions above. You may also include additional comments for the author, including concerns about dual publication, research ethics, or publication ethics. (Please upload your review as an attachment if it exceeds 20,000 characters)

Reviewer #1: In this R3 draft the authors have made some constructive changes in response to my earlier reviews, but there are still some areas where improvement would be good.

They have included some reference to the univariate Fischer’s exact tests of differences in Table 1’s work variables and their chosen covariates in the first paragraph of the Results section, but I believe they need to include more details – i.e., in men with CACS >100 the 38% percent with high strain job was NOT significantly different from the 25% in men with CACS of 0. In contrast, Table 1 shows that in men with CASC > 100 or 1-99 both % Ever smoker and Metabolic syndrome were significantly elevated compared to men with CACS of 0; and in women those with CACS >100 had significantly higher % with No University and living in Low SES area than those with CADS 0, and those with CACS 1-99 had significantly higher % with metabolic syndrome. The higher % in passive work conditions in those women with CACS > 100 is NOT significant. I believe that the significant associations between CACS levels and % with Metabolic syndrome and Ever smoker in men and between CACS levels and No university and low status SES area and Metabolic syndrome in women should be reported in more detail – e.g., 83% of women with CACS >100 had No university vs only 48% of women with CACS of 0, and give the P-values (e.g., P<0.05) for each significant one.

They say they have moderated the conclusion to take into account that none of the tests of associations between job stress and other measures with CACS were significant, but the conclusion of the R3 draft is the same as that of the R2 draft. I suggest that they do not assert “Our results indicate…” – I suggest the say instead, “Our results suggest the possibility that exposure ….” They should also include some details of the significant associations in Table 1 of job stress measures and covaried factors with CACS in the conclusion.

In response to my suggestion that they test whether factors they found associated with increased CACS in Table 1 are also associated with job stress measures, they say they have “checked this and none of the adjustment factors was significantly related to high strain jobs, among neither men nor women.” And “However we will not include this in the results section since this is not our research question.” I disagree and recommend they include that these associations were tested and all were nonsignificant.

They also say they did not explore associations between components of the metabolic syndrome that are accounting for the association between metabolic syndrome and increased CACS in both men and women because “This study was not an exploratory study…We do not believe it is correct to set up a new research question within the same study when finding out that we have insignificant results.” I also disagree with this argument – indeed, when one has insignificant results, it can be considered appropriate to carry out additional analyses to try to contribute something more than failure to confirm their hypothesis that job stress would be associated with increased CACS.

In response to my concern that it is not clear how doing stratified analyses of job stress associations with CACS in men with MetSyn vs those with no MetSyn addresses the question of mediation of job stress impact on CACS, they say they therefore did the stratified analyses, but say nothing regarding implications of these analyses being nonsignifcant.

**********

7. PLOS authors have the option to publish the peer review history of their article (what does this mean?). If published, this will include your full peer review and any attached files.

If you choose “no”, your identity will remain anonymous but your review may still be made public.

**Do you want your identity to be public for this peer review?** For information about this choice, including consent withdrawal, please see our Privacy Policy.

Reviewer #1: No

[NOTE: If reviewer comments were submitted as an attachment file, they will be attached to this email and accessible via the submission site. Please log into your account, locate the manuscript record, and check for the action link "View Attachments". If this link does not appear, there are no attachment files.]

While revising your submission, please upload your figure files to the Preflight Analysis and Conversion Engine (PACE) digital diagnostic tool, https://pacev2.apexcovantage.com/. PACE helps ensure that figures meet PLOS requirements. To use PACE, you must first register as a user. Registration is free. Then, login and navigate to the UPLOAD tab, where you will find detailed instructions on how to use the tool. If you encounter any issues or have any questions when using PACE, please email PLOS at figures@plos.org. Please note that Supporting Information files do not need this step.

# Author comment: Author response to Decision Letter 3

**DOI:** 10.1371/journal.pone.0252192.r008

29 Apr 2021

29th of April 2021, Gothenburg

Response to reviewers. 

We have marked all of our changes in yellow in the manuscript. 

PLOS ONE

Journal Requirements:

Please review your reference list to ensure that it is complete and correct. If you have cited papers that have been retracted, please include the rationale for doing so in the manuscript text, or remove these references and replace them with relevant current references. Any changes to the reference list should be mentioned in the rebuttal letter that accompanies your revised manuscript. If you need to cite a retracted article, indicate the article’s retracted status in the References list and also include a citation and full reference for the retraction notice.

Thank you for your comment, regarding reference 27, we have now added “Corrected version” after the reference. We have also translated reference 4, 9 and 10 to English. 

[Note: HTML markup is below. Please do not edit.]

Reviewers' comments:

Reviewer's Responses to Questions

Comments to the Author

Reviewer #1: In this R3 draft the authors have made some constructive changes in response to my earlier reviews, but there are still some areas where improvement would be good.

They have included some reference to the univariate Fischer’s exact tests of differences in Table 1’s work variables and their chosen covariates in the first paragraph of the Results section, but I believe they need to include more details – i.e., in men with CACS >100 the 38% percent with high strain job was NOT significantly different from the 25% in men with CACS of 0. In contrast, Table 1 shows that in men with CASC > 100 or 1-99 both % Ever smoker and Metabolic syndrome were significantly elevated compared to men with CACS of 0; and in women those with CACS >100 had significantly higher % with No University and living in Low SES area than those with CADS 0, and those with CACS 1-99 had significantly higher % with metabolic syndrome. The higher % in passive work conditions in those women with CACS > 100 is NOT significant. I believe that the significant associations between CACS levels and % with Metabolic syndrome and Ever smoker in men and between CACS levels and No university and low status SES area and Metabolic syndrome in women should be reported in more detail – e.g., 83% of women with CACS >100 had No university vs only 48% of women with CACS of 0, and give the P-values (e.g., P<0.05) for each significant one. 

We apologize for causing a misunderstanding. It is only the covariates metabolic syndrome, no university education, ever smoker and socioeconomic area that we have previously marked with an asterisk in table 1, if it differed significantly, we have not marked high strain job, passive job, active job or low strain job. The reason is that these are unadjusted results and can be misleading as we later in the manuscript present the prevalence ratios between CACS and passive/low/high strain and active job, which are adjusted and therefor more robust results. But we have now also presented in table 1 which of the job types that differed significantly. We have also adjusted the text about table 1 in the results section, first paragraph and added only significant results from table 1 in the text. But please note that the results in table 1 are descriptive and not adjusted. 

They say they have moderated the conclusion to take into account that none of the tests of associations between job stress and other measures with CACS were significant, but the conclusion of the R3 draft is the same as that of the R2 draft. I suggest that they do not assert “Our results indicate…” – I suggest the say instead, “Our results suggest the possibility that exposure ….” 

We have modified the conclusion according to your constructive suggestion, thank you. 

They should also include some details of the significant associations in Table 1 of job stress measures and covaried factors with CACS in the conclusion.

In Table 1, we present the covariates that we adjust our main results with. The results in Table 1 are unadjusted for age, and not part of our research question so we do not consider it a part of the conclusion. 

In response to my suggestion that they test whether factors they found associated with increased CACS in Table 1 are also associated with job stress measures, they say they have “checked this and none of the adjustment factors was significantly related to high strain jobs, among neither men nor women.” And “However we will not include this in the results section since this is not our research question.” I disagree and recommend they include that these associations were tested and all were nonsignificant.

We have added a sentence in the results section, first paragraph, according to your recommendation. 

They also say they did not explore associations between components of the metabolic syndrome that are accounting for the association between metabolic syndrome and increased CACS in both men and women because “This study was not an exploratory study…We do not believe it is correct to set up a new research question within the same study when finding out that we have insignificant results.” I also disagree with this argument – indeed, when one has insignificant results, it can be considered appropriate to carry out additional analyses to try to contribute something more than failure to confirm their hypothesis that job stress would be associated with increased CACS.

We appreciate your interest in which factors affect CACS but we do not consider it appropriate to change our research question and we do not have enough statistical power to do the suggested analyses. 

In response to my concern that it is not clear how doing stratified analyses of job stress associations with CACS in men with MetSyn vs those with no MetSyn addresses the question of mediation of job stress impact on CACS, they say they therefore did the stratified analyses, but say nothing regarding implications of these analyses being nonsignifcant.

We have added a part in the discussion section, third paragraph, on this topic, thank you for your remark. 

________________________________________

Attachment Submitted filename: resprevapr21cacserikssonsubm.docx Click here for additional data file.

# Peer review: Decision Letter 4

**DOI:** 10.1371/journal.pone.0252192.r009

**Authors:** Xianwu Cheng

12 May 2021

Psychosocial job exposure and risk of coronary artery calcification

PONE-D-20-20980R4

Dear Dr. Eriksson

We’re pleased to inform you that your manuscript has been judged scientifically suitable for publication and will be formally accepted for publication once it meets all outstanding technical requirements.

Within one week, you’ll receive an e-mail detailing the required amendments. When these have been addressed, you’ll receive a formal acceptance letter and your manuscript will be scheduled for publication.

An invoice for payment will follow shortly after the formal acceptance. To ensure an efficient process, please log into Editorial Manager at http://www.editorialmanager.com/pone/, click the 'Update My Information' link at the top of the page, and double check that your user information is up-to-date. If you have any billing related questions, please contact our Author Billing department directly at authorbilling@plos.org.

If your institution or institutions have a press office, please notify them about your upcoming paper to help maximize its impact. If they’ll be preparing press materials, please inform our press team as soon as possible -- no later than 48 hours after receiving the formal acceptance. Your manuscript will remain under strict press embargo until 2 pm Eastern Time on the date of publication. For more information, please contact onepress@plos.org.

Kind regards,

Xianwu Cheng, M.D., Ph.D., FAHA

Academic Editor

PLOS ONE

Additional Editor Comments (optional):

Reviewers' comments:

Reviewer's Responses to Questions

**Comments to the Author**

1. If the authors have adequately addressed your comments raised in a previous round of review and you feel that this manuscript is now acceptable for publication, you may indicate that here to bypass the “Comments to the Author” section, enter your conflict of interest statement in the “Confidential to Editor” section, and submit your "Accept" recommendation.

Reviewer #1: All comments have been addressed

**********

2. Is the manuscript technically sound, and do the data support the conclusions?

The manuscript must describe a technically sound piece of scientific research with data that supports the conclusions. Experiments must have been conducted rigorously, with appropriate controls, replication, and sample sizes. The conclusions must be drawn appropriately based on the data presented. 

Reviewer #1: Yes

**********

3. Has the statistical analysis been performed appropriately and rigorously? 

Reviewer #1: Yes

**********

4. Have the authors made all data underlying the findings in their manuscript fully available?

The PLOS Data policy requires authors to make all data underlying the findings described in their manuscript fully available without restriction, with rare exception (please refer to the Data Availability Statement in the manuscript PDF file). The data should be provided as part of the manuscript or its supporting information, or deposited to a public repository. For example, in addition to summary statistics, the data points behind means, medians and variance measures should be available. If there are restrictions on publicly sharing data—e.g. participant privacy or use of data from a third party—those must be specified.

Reviewer #1: No

**********

5. Is the manuscript presented in an intelligible fashion and written in standard English?

PLOS ONE does not copyedit accepted manuscripts, so the language in submitted articles must be clear, correct, and unambiguous. Any typographical or grammatical errors should be corrected at revision, so please note any specific errors here.

Reviewer #1: Yes

**********

6. Review Comments to the Author

Please use the space provided to explain your answers to the questions above. You may also include additional comments for the author, including concerns about dual publication, research ethics, or publication ethics. (Please upload your review as an attachment if it exceeds 20,000 characters)

Reviewer #1: (No Response)

**********

7. PLOS authors have the option to publish the peer review history of their article (what does this mean?). If published, this will include your full peer review and any attached files.

If you choose “no”, your identity will remain anonymous but your review may still be made public.

**Do you want your identity to be public for this peer review?** For information about this choice, including consent withdrawal, please see our Privacy Policy.

Reviewer #1: No

# Editor report: Acceptance letter

**DOI:** 10.1371/journal.pone.0252192.r010

**Authors:** Xianwu Cheng

17 May 2021

PONE-D-20-20980R4 

Psychosocial job exposure and risk of coronary artery calcification 

Dear Dr. Eriksson:

I'm pleased to inform you that your manuscript has been deemed suitable for publication in PLOS ONE. Congratulations! Your manuscript is now with our production department. 

If your institution or institutions have a press office, please let them know about your upcoming paper now to help maximize its impact. If they'll be preparing press materials, please inform our press team within the next 48 hours. Your manuscript will remain under strict press embargo until 2 pm Eastern Time on the date of publication. For more information please contact onepress@plos.org.

If we can help with anything else, please email us at plosone@plos.org. 

Thank you for submitting your work to PLOS ONE and supporting open access. 

Kind regards, 

PLOS ONE Editorial Office Staff

on behalf of

Associate Prof. Xianwu Cheng 

Academic Editor

PLOS ONE
